# Ecotoxicity to Freshwater Organisms and Cytotoxicity of Nanomaterials: Are We Generating Sufficient Data for Their Risk Assessment?

**DOI:** 10.3390/nano11010066

**Published:** 2020-12-30

**Authors:** Tatiana Andreani, Verónica Nogueira, Ana Gavina, Saul Fernandes, José Luís Rodrigues, Vera V. Pinto, Maria José Ferreira, Amélia M. Silva, Carlos M. Pereira, Ruth Pereira

**Affiliations:** 1 Centro de Investigação em Química da Universidade do Porto, CIQUP & Department of Chemistry and Biochemistry, Faculty of Sciences of the University of Porto, Rua do Campo Alegre, 4169-007 Porto, Portugal; cmpereir@fc.up.pt; 2 CITAB—Centre for Research and Technology of Agro-Environmental and Biological Sciences, University of Trás-os-Montes e Alto Douro, UTAD, 5000-801 Vila Real, Portugal; amsilva@utad.pt; 3 GreenUPorto—Sustainable Agrifood Production Research Centre & Department of Biology, Faculty of Sciences of the University of Porto, Rua do Campo Alegre s/n, 4169-007 Porto, Portugal; ana.gavina@fc.up.pt (A.G.); saulsimao@gmail.com (S.F.); 4 Interdisciplinary Centre of Marine and Environmental Research (CIIMAR), University of Porto, Terminal de Cruzeiros do Porto de Leixões, Av. General Norton de Matos s/n, 4450-208 Matosinhos, Portugal; veronica.nogueira@fc.up.pt; 5 Centro Tecnológico do Calçado de Portugal, Rua de Fundões—Devesa Velha, 3700-121 São João Madeira, Portugal; jose.rodrigues@ctcp.pt (J.L.R.); Vera.pinto@ctcp.pt (V.V.P.); MJose.Ferreira@ctcp.pt (M.J.F.); 6 Department of Biology and Environment, University of Trás-os-Montes e Alto Douro, UTAD, Quinta de Prados, P-5000-801 Vila Real, Portugal

**Keywords:** metal oxide nanomaterials, toxicity, *Raphidocelis subcapitata*, *Daphnia magna*, *Lemna minor*, human cell lines, PNEC, HC_5_, risk assessment

## Abstract

The aim of the present study was to investigate the eco-cytotoxicity of several forms of nanomaterials (NM), such as nano-CuO, nano-TiO_2_, nano-SiO_2_ and nano-ZnO, on different aquatic species (*Raphidocelis subcapitata*, *Daphnia magna* and *Lemna minor*) following standard protocols and on human cell lines (Caco-2, SV-80, HepG2 and HaCaT). Predicted no-effect concentrations (PNEC) or hazard concentrations for 5% of the species (HC_5_) were also estimated based on the compilation of data available in the literature. Most of the NM agglomerated strongly in the selected culture media. For the ecotoxicity assays, nano-CuO and nano-ZnO even in particle agglomeration state were the most toxic NM to the freshwater organisms compared to nano-TiO_2_ and nano-SiO_2_. Nano-ZnO was the most toxic NM to *R. subcapitata* and *D. magna*, while nano-CuO was found to be very toxic to *L. minor*. Nano-CuO was very toxic to Caco-2 and HepG2 cells, particularly at the highest tested concentrations, while the other NM showed no toxicity to the different cell lines. The HC_5_ and PNEC values are still highly protective, due to data limitations. However, the present study provides consistent evidence of the potential risks of both nano-CuO and nano-ZnO against aquatic organisms and also their effects on public health.

## 1. Introduction

The industrial applications of nanomaterials (NM) have gained great significance by increasing the efficacy and stability of several market products [1,2]. Nevertheless, apart from the rapid advent of nanotechnology in a large number of industries, the toxicity of these new entities is still unknown and there is a general apprehension in society regarding the human and environmental risks associated with NM exposure [3]. The concern is not only about the possible toxic effects of NM on human health during the use of NM-containing products but also the impact on ecosystems at the end of their lifecycle and after the disposal of nano-based products in the environment [4,5,6]. In fact, although some products containing NM are currently available in the market, information about their toxicological data are only now emerging.

NM can be released from manufactured products into water resources during the manufacturing processes, during their life through usage and washing and/or by the elimination of the products at the end of their lifecycle [7,8,9]. The effects of these products containing NM on public health and biota (aquatic/terrestrial organisms) during their lifecycle are inevitable and thus it is essential to assess what these effects are.

The diversity of NM and their physicochemical properties, as well as their behavior under environmental conditions, are the reason for several knowledge gaps. Although there are numerous studies correlating the impact of NM with their physicochemical properties (e.g., particle size, surface charge, surface coating, shape and ion dissolution) [10,11,12,13,14], most in vitro and in vivo data from NM toxicity assessments are contradictory, since toxic and non-toxic effects are often reported apparently for the same NM (for example, nano-TiO_2_), but, in fact, the great majority of studies were conducted with different NM (e.g., nano-TiO_2_ can be commercially acquired or synthesized in the lab; it can be in anatase or in rutile form, it can have different particle size/surface charge/morphology, and it can be functionalized or not). Therefore, the conclusions about the hazardous nature of NM are poorly supported and/or frequently reported as ambiguous due to the wide variety of NM types being tested. Additionally, changes in NM physicochemical properties, when exposed to a particular aqueous medium, may lead to the formation of agglomerates/aggregates in aqueous suspensions, especially in those with high ionic strength, which may alter the toxicological profile.

Most existing data concerning the toxicity of NM show a size-dependent effect, in which toxicity increases with decreasing particle size [15,16]. Small particles possess higher surface area-to-volume ratio in comparison to larger particles, leading to an expected increase in particle reactivity and toxicity. However, considering that NM can form aggregates/agglomerates when dispersed into high-ionic-strength solutions (e.g., culture media) [17,18], as well as in the presence of organic matter [19], the agglomeration state of NM should not be neglected in toxicity testing. For example, the evaluation of the toxicity of NM dispersed in aqueous suspensions, or in a particular organic solvent that presents low values of average particle size and low polydispersity index, may not be representative of the state of NM in real environmental scenarios (culture media), where particle aggregation/agglomeration can occur. Several works have correlated the toxicity of NM to a particular organism or human cell line with the NM’s small particle size, without evaluating their real physical properties in the exposure medium, which may lead to misinterpretation and illusion about the effects of NM. In our previous work, we showed that even in large agglomerates, metal NM (silver and cooper) can be hazardous to aquatic biota and human cells at concentrations ≤ 20 mg L^−1^ [17]. In this context, not only do the physical properties of each NM play an important role in the toxicological assays, but so do the chemical composition, the behavior and fate of NM under environmental conditions; and mainly the surface interactions between NM with biological membranes must also be taken into account.

Based on these considerations, the present study focuses on the characterization and the toxicity evaluation of NM widely used in consumer products, such as nano-CuO, nano-TiO_2_, nano-ZnO and nano-SiO_2_. Due to their biocidal characteristics, nano-TiO_2_ and nano-ZnO have been extensively explored in several industrial classes of products, including medical tools, cosmetics, food and fabric coatings to control bacteria and fungi growth [20]. In addition, because of their ability to filter UV-A and UV-B radiation, nano-TiO_2_ and nano-ZnO have been used in sunscreen formulations [21]. Nano-TiO_2_ is also applied in toothpaste to provide whiteness and opacity [22]. Nano-CuO has great potential in thermal conductivity [23], while nano-SiO_2_ is a popular anticaking agent widely used in food products [24]. Although the literature suggests that these NM are among the most studied, an exhaustive bibliographic search has shown that, in fact, although there is a large amount of toxicity data available for NM, there are also many differences in their physical properties, as well as in the experimental conditions of toxicity assays performed to evaluate their impact on the environment and public health. The lack of coherence in toxicity data regarding NM is in part due to differences in NM within the same category (commercial versus lab-synthesized NM, and several methods of synthesis), differences in susceptibility among the test organisms/cell lines and great diversity in the experimental conditions (e.g., dispersion medium, exposure time and pH). Therefore, the present study was designed to investigate: (i) existing data for the metal oxide NM used in this study, in order to testify to the novelty of our results and to provide evidence that we are far from having sufficient data to support the risk assessment for the great diversity of NM that are being manufactured; (ii) the physicochemical properties of the different commercial metal oxide NM when suspended in the culture media of distinct test organisms in order to reflect adequately the exposure modes; (iii) the effects of NM on aquatic organisms (microalgae *Raphidocelis subcapitata*, crustaceans *Daphnia magna* and aquatic plants *Lemna minor*) and (iv) the effects of NM on human cellular lines derived from intestine (Caco-2), liver (HepG2), skin (HaCaT) and lung (SV-80), representing the main human exposure pathways.

## 2. Materials and Methods

### 2.1. Nanomaterials, Chemicals and Reagents

The commercial NM used in the present study were obtained from PlasmaChem GmbH Company (Berlin, Germany) and are listed as follows: zinc oxide powder (ZnO, particle size of 25 nm), hydrophilic TiO_2_ powder stabilized by HNO_3_ (TiO_2_, particle size ranging from 4 to 8 nm, anatase phase), fumed hydrophilic SiO_2_ nanopowder (particle size ranging from 7 to 14 nm), SiO_2_ in a 30% aqueous suspension (average particle size of 10 nm) and CuO (average particle size of 40 nm).

Fetal bovine serum (FBS), Dulbecco’s Modified Eagle’s Medium (DMEM), antibiotics solution (composed by penicillin and streptomycin), L-glutamine, 0.05% trypsin–ethylenediamine tetraacetic acid (EDTA) solution and Alamar Blue^®^ (AB) reagent were obtained from ThermoFisher Scientific^®^ (Alfagene^®^, Carcavelos, Portugal). Ultrapure water was produced by the Milli Q^®^ water purification process (18.2 MΩ cm, Millipore, Darmstadt, Germany).

### 2.2. Suspensions and Characterization of NM

To verify the NM behavior under experimental conditions similar to those used in the toxicity studies, three intrinsic properties of NM were evaluated: the mean particle size, the polydispersity index (PI) and surface charge. For this purpose, the suspensions were prepared at 20 mg L^−1^ (in respective culture media), the highest concentration used for algae *R. subcapitata* (Woods Hole MBL), *D. magna* (ASTM hard medium), *L. minor* (Steinberg medium) and human cell lines (DMEM), followed by sonication for 30 min in an ultrasonic bath (Ultrasonic Cleaner, SONICA^®^, Soltec, Milan, Italy).

Dynamic light scattering (DLS) measurements were performed on a W130i instrument (Avid Nano, High Wycombe, UK), at 20 °C, to analyze the mean particle size and the PI of NM by using a quartz cell (Hellma^®^ Analytics, 111-QS, 10 mm × 10 mm, Jena, Germany). The hydrodynamic particle size was calculated by the cumulant method. The surface charge of NM was recorded by measuring the zeta potential (ZP) in a Zetasizer Nano ZS equipment (Malvern Instruments Inc., Malvern, UK) at 20 °C. All the measurements were performed in triplicate.

The determination of copper, zinc and titanium concentrations released from NM into different culture media was performed by inductively coupled plasma optical emission spectrometry (ICP-OES) (Horiba Jobin Yvon, Activa M, Longjumeu, France). The suspensions were prepared at 20 mg L^−1^ according to the procedure described above, centrifuged at 6000 rpm for 30 min, followed by the acidification of the supernatant with HNO_3_ 1% (*v*/*v*).

### 2.3. Maintenance of Organism Cultures and Human Cell Lines

Freshwater green microalgae *R. subcapitata* were maintained in our laboratory and cultivated in 4-L glass flasks containing sterile medium (Woods Hole MBL) [25] in a growth chamber under continuous light, at 20 ± 2 °C, 6000–10,000 Lux (cool white light fluorescent lamps), with constant aeration.

*L. minor* plants were originally collected from the Botanic Garden (University of Porto) and the best fitted plants were washed with distilled water and maintained in an Erlenmeyer containing Steinberg growth medium [26] under 16 h^L^:8 h^D^, at 20 ± 2 °C and light intensity of 6000–10,000 Lux, provided by cool white light fluorescent lamps. The culture medium was renewed once a week.

Adult crustaceans *D. magna* were cultured in the glass flasks (~30 animals/800 mL) containing ASTM hard water [27] supplemented with organic extract, vitamins and suspension of green microalgae *R. subcapitata* provided for food. The medium was replaced three times per week and the animals were maintained at a constant temperature (20 ± 2 °C) with a natural light–dark cycle.

The human cell lines Caco-2 (derived from human colorectal adenocarcinoma), SV-80 (human lung fibroblast) and HaCaT (human keratinocytes) were purchased from Cell Line Services (CLS; Eppelheim, Germany) and HepG2 (human hepatic carcinoma; American Type Culture Collection (ATCC) N° HB-8065^TM^) were kindly offered by Prof. Carlos Palmeira from Coimbra University, Portugal. Cells were cultured in DMEM containing FBS (10%, *v*/*v*), penicillin (100 U mL^−1^), streptomycin (100 µg mL^−1^) and 1 mM L-glutamine. All cell cultures were maintained in an incubator at 37 °C under a humidified atmosphere of 5% CO_2_. The culture medium was replaced every 2 days.

### 2.4. Ecotoxicity Assays

#### 2.4.1. *R. Subcapitata* Growth Inhibition Test

The microalgae growth inhibition tests were performed according to OCDE 201 protocol [28]. The stock culture was previously prepared by adding 20 mL of algae inoculum to 50 mL of MBL medium and incubated under 16^L^:8^D^, at 21 ± 2 °C, during 72 h. After this period, the density of algae in the original stock suspension was determined through optical microscopy on the Neubauer chamber, diluted in MBL, to obtain an initial density of 10^4^ cells mL^−1^, and seeded in 24-well microplates.

For the toxicity assay, NM were dispersed into MBL medium (please see Section 2.3). The tested concentrations ranged from 0 to 20 mg L^−1^ (dilution factor of 1.6) for nano-CuO, hydrophilic nano-TiO_2_, hydrophilic nano-SiO_2_ and SiO_2_ (30% aq. suspension). For nano-ZnO, the tested concentrations ranged from 0 to 5 mg L^−1^ (dilution factor of 1.4). For the assay, each concentration was tested in triplicate, while the control group was tested in sextuplicate. The microplates were maintained under the same conditions as described above for the culture and homogenized manually twice a day with a micropipette to avoid algal agglomerates. The cell density in each replica was counted through an optical microscope using a Neubauer chamber. The microalgae growth inhibition (in percentage) for each concentration of NM was determined in relation to the control group (without NM).

#### 2.4.2. *L. Minor* Growth Inhibition Test

*L. minor* growth test was based on the OECD guideline 221 [26]. Three colonies with three fronds each were transferred to test vessels containing a volume of 60 mL (only Steinberg medium for the control group or Steinberg medium plus NM for the treatment groups) under axenic conditions. For each test vessel, the number of colonies and the frond number were the same. Plants were exposed to suspensions of NM at concentrations ranging from 0 to 20 mg L^−1^ with a dilution factor of 1.6, except for nano-CuO, in which the tested concentrations ranged from 0 to 1.9 mg L^−1^, also applying a dilution factor of 1.6. Regarding nano-CuO, we initially tested a concentration range of 0 to 20 mg L^−1^. However, a severe decrease in *L. minor* growth rate was observed with concentrations above 1.9 mg L^−1^.

The toxicity test for each concentration and control group was performed in triplicate and maintained in the same conditions as described for the stock culture maintenance. Three colonies with tree fronds were collected from the stock culture plants and dried at 60 °C overnight to provide the initial dry weight of the plants. The effect of NM was evaluated according to the growth rate of *L. minor* calculated based on the number of fronds and on the dry weight of the fronds after 7 days of exposure.

#### 2.4.3. *D. Magna* Immobilization Test

Acute toxicity test using *D. magna* was performed according to OECD guideline 202 [29]. The toxicity test was assessed using five *D. magna* neonates (age ˂ 24 h) per glass tube (i.e., per replicate) containing 25 mL ASTM hard water medium with different concentrations of the NM in suspension. Nano-TiO_2_, hydrophilic nano-SiO_2_, nano-SiO_2_ (30% aq. suspension) were tested at concentrations ranging from 0 to 20 mg L^−1^ (dilution factor of 1.6). Nano-CuO was tested at concentrations ranging from 0 to 7.3 (dilution factor of 1.4), while nano-ZnO was tested from 0 to 1.9 mg L^−1^ (dilution factor of 1.6). Each concentration was tested in quadruplicate. The tubes were incubated under the same conditions as described above for the culture maintenance. After 24 h and 48 h of exposure to suspensions of NM, the immobilization of the organisms was visually inspected, and the number of immobilized organisms was counted, taking into account their ability to swim freely within 15 s.

### 2.5. Cytotoxicity Tests with Human Cell Lines

The assessment of NM cytotoxicity was performed using the AlamarBlue^®^ (AB) reagent as an indicator of cell proliferation and viability [30]. Viable cells are able to reduce AB (resazurin, blue color) to resorufin (pink color) and therefore the oxidation/reduction reaction can be quantitatively measured by reading the absorbance of culture media. Cell’s viability correlates with the percentage of AB reduction, which can be determined as previously reported in Andreani et al. (2014) [30].

For the procedure, cells were detached using a trypsin–EDTA solution at 37 °C for 7 min, counted using an automated cell counter (TC10—, BIORAD, Amadora, Portugal) and diluted in culture medium (with FBS) to adjust the cellular density to 5 × 10^4^ cell mL^−1^. Then, cells were seeded in 96-well plates (100 μL/well) and cultured for 24 h to allow cell attachment. After this, the culture medium was removed and replaced by FBS-free culture medium containing the dispersions of NM at final concentrations of 0.0, 5.0, 10, 15 and 20 mg L^−1^ and incubated for 24 or 48 h, at 37 °C and 5% CO_2_ atmosphere. The toxicity of each concentration was evaluated in quadruplicate and cells not exposed to NM were used as negative controls. Subsequent to the incubation time (24 or 48 h), the culture media of the control group and of the treatment groups were removed and AB solution (10% (*v*/*v*) prepared in fresh FBS-free culture medium) was added to each well (100 µL/well), followed by an additional incubation for 4 h (controlled humidity, 37 °C and 5% CO_2_). Then, a Labsystem Multiskan EX microplate reader (LabX, Midland, ON, Canada) was used to read the absorbance of each well at 570 and 620 nm. As stated by Doktorovová et al. (2014), a biologically inert NM is one that elicits a cell viability equal to or higher than 70% [31].

### 2.6. Statistical Analysis

For all experiments, the data were reported as mean ± standard deviation (SD). Statistically significant differences between treatments for each assay and parameter assessed were tested by univariate analysis of variance (ANOVA) with subsequent Dunnett’s post hoc test to determine the differences between each treatment and the control group. No observed effect concentrations (NOEC) and low observed effect concentrations (LOEC) were determined based on the previously described analysis. The EC50 and EC20 values were determined by applying non-linear least squares regression analysis using STATISTICA^®^ 7.0 software (StatSoft, Inc., Tulsa, OK, USA). For *D. magna* immobilization assay, lethal concentrations (LC_50_) were estimated after fitting a Probit regression to the binary data [32] using SPSS^®^ Statistics software version 25 (Chicago, IL, USA).

In order to provide predicted no-effect concentrations (PNEC) or hazard concentrations for 5% of the species (HC_5_) for the NM under evaluation and for the freshwater organisms, deterministic and probabilistic approaches were followed by applying assessment factors or by obtaining species sensitivity distributions (SSD), respectively [33,34]. For the estimation of PNEC and HC_5_ values, data from the literature were selected (Table S1) when they were reported for the same NM tested in this study, regarding its chemical nature, independently of the particle size reported by the manufacturer. The SSD were generated only for two NM, for which EC_50_ values were available (LOEC and NOEC values were not used, since they are not statistical estimations), for 6 different species by fitting a linearized log-normal model to data, with the USEPA SSD Generator V1 (USEPA, Boston, MA, USA), available at https://www.epa.gov/caddis-vol4/caddis-volume-4-data-analysis-download-software. When more than one data point was available for the same species and endpoint, a geometric mean was calculated. For PNEC or HC_5_ estimation, a bibliographic search was carried out in May 2020 by using the ISI WEB of Science database and by combining the following keywords: TS = (nano*) AND TS = (titanium dioxide) AND TS = (toxicity or Ecotoxic*) AND TS = (freshwater). The search was repeated for silicon dioxide, silica dioxide, zinc oxide and copper oxide. All the papers found were analyzed and it was decided to use only those that tested the toxicity of NM following standard guidelines for ecotoxicological assays with freshwater species.

## 3. Results and Discussion

### 3.1. Behavior of NM in Culture Media

The behavior and physical characterization of NM suspended in the different culture/test media were investigated by DLS and electrophoretic mobility. The characterization of NM in regard to hydrodynamic particle size, PI and ZP is depicted in Table 1. Data from physicochemical parameters clearly showed the formation of particle agglomerates. Strong agglomeration was observed especially for dispersions of nano-CuO in ASTM, Steinberg and DMEM test media, for nano-TiO_2_ in ASTM and DMEM media and for nano-ZnO in Steinberg medium, achieving average hydrodynamic sizes out of the range of the equipment measurements. In opposition, nano-SiO_2_ (aq) dispersed in MBL or ASTM was revealed to have a particle size closer to that supplied by the manufacturer (~10 nm), with a homogenous size distribution, as shown by the low PI values. From the ZP measurements, all NM presented negative surface charges when suspended in the different culture media. The low surface charge (absolute values) of NM correlates directly with the drastic increase in the average hydrodynamic size of NM, suggesting the high instability of the suspensions. For all NM, the absolute highest ZP values were observed when NM were dispersed into MBL (>|20 mV|), showing that these dispersions may present moderate stability compared to the others. However, as can be observed from the data, although the hydrophilic nano-SiO_2_ dispersed into MBL showed a high PI (0.86), the ZP value is also high. The same occurred with nano-SiO_2_ (aq) when dispersed in ASTM, showing a decrease in ZP values in comparison with MBL suspensions (from −27.3 to −15.3 mV), despite the decrease in the PI value. Therefore, although NM dispersions with |ZP| > 30 mV are considered stable, ZP measurements only refer to the electrostatic effect (repulsive forces) between particles but do not supply any information on the attractive van der Waals forces, which may also play a role in nanoparticle stability. According to the well-known classic theory of Derjaguin, Landau, Verwey and Overbeek (DLVO), the particle stabilization can be explained as a superposition of the repulsive electrostatic potential and the attractive van der Waals forces [35]. Thus, in high-ionic-strength environments, the particle agglomeration can be attributed to a decrease in repulsive forces due to a compression of the electrical double layer and an increase in van der Waals interactions [36].

As reported by several works, the particle size can be affected by environmental pH, ionic strength and by the presence of plasma proteins [37,38]. Although there are numerous types of NM, various homogenization techniques (sonication, magnetic stirring or vortex) and homogenization times, our results were consistent with others reported in the literature. As described above, in the present study, nano-TiO_2_ (stabilized with HNO_3_, anatase phase) showed severe particle agglomeration when dispersed in the different culture media after sonication in a water bath. In accordance with our results (Table 1), Nogueira et al. (2015) also showed high particle agglomeration (high Z-Ave and PI) concomitantly with a low ZP after the suspension of nano-TiO_2_ (anatase phase) in ASTM, MBL and Steinberg media through magnetic stirring [18], suggesting that sonication does not bring great benefits in terms of NM suspension stability. In addition, these authors also reported that nano-TiO_2_ in MBL showed lower particle agglomeration and higher ZP values when compared with the dispersion of nano-TiO_2_ in ASTM or Steinberg media. Although nano-TiO_2_ type P25 (combination of anatase and rutile phases) is different from the nano-TiO_2_ tested in this study (anatase), a study conducted by Ji et al. (2010) also reported that nano-TiO_2_ type P25 suspended in DMEM by sonication (in a water bath) and by vortex showed high particle agglomeration, obtaining a hydrodynamic diameter of 843 nm, a PI of 0.021 and a ZP of –7.4 mV [36]. In this study (Table 1), DMEM was in fact the media causing the highest particle agglomeration for almost all the tested NM. Contrarily to our data (Table 1), the agglomeration of particles observed by Ji et al. (2010) was not accompanied by an increase in PI value, indicating that even in the agglomeration state, the size of the particles remains homogeneous.

The concentration of free ions in the test medium is an important parameter that may affect the toxic behavior of metallic NM [39]. According to the free ion quantification, a direct connection between the free metal ions in the culture media and the particle agglomeration was observed for all NM dispersions (Table 1). Results indicate that, for all tested NM, the highest concentrations of dissolved metal ions in the culture media were also detected when high particle agglomeration was recorded. In addition, the highest concentrations of soluble metal ions were found in Steinberg medium, for all NM, while the lowest concentrations were detected in MBL for nano-CuO and nano-ZnO and in DMEM for nano-TiO_2_ (Table 1). Although there was a direct correlation between high particle agglomeration and high concentration of free metal ions, this result can be likely ascribed to the pH of the different culture media used in the present study. The low pH of Steinberg medium (around 5.5) may contribute to the higher release of metal ions compared with the other media, since the pH levels in MBL and ASTM are around 7.2 and 8.0, respectively. In addition, although high particle agglomeration was observed, it is possible that there were small particles contributing to the increase in free metal ion concentration. This hypothesis is supported by the high PI values recorded, which point towards polydisperse suspensions.

### 3.2. Effect of NM on R. Subcapitata Growth

**Figure 1:**
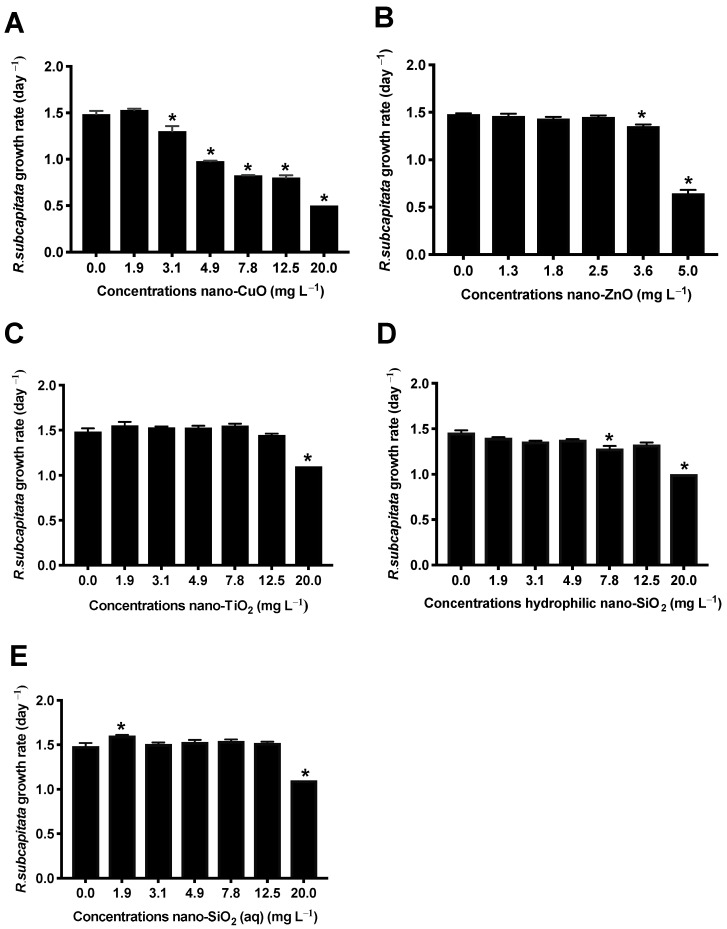
*R. subcapitata* growth rate after 72 h of exposure to nano-CuO (**A**), nano-ZnO (**B**), nano-TiO_2_ (**C**), hydrophilic nano-SiO_2_ (**D**), nano-SiO_2_ (aq) (**E**). The experiments were conducted in triplicate and the results are shown as the mean ± standard deviation (ANOVA followed by Dunnett’s test, * *p* ˂ 0.05 compared to control).

After 72 h of exposure, nano-CuO induced high toxicity to algae *R. subcapitata*, with an EC_50_ of 12.77 mg L^−1^ (Figure 1A). Nevertheless, nano-ZnO was the most toxic NM to *R. subcapitata*, depicting an EC_50_ = 4.86 mg L^−1^ (Figure 1B, Table 2). The growth curves demonstrated that, for nano-TiO_2_, a significant difference in the algae growth rate compared with the control group was only observed at 20 mg L^−1^ (*p* < 0.05), where the growth inhibition was 27% (Figure 1C). Similar results were obtained for hydrophilic nano-SiO_2_ and nano-SiO_2_ (aq), for which the percentage of algae growth inhibition at 20 mg L^−1^ was 30% and 23.9%, respectively (Figure 1D,E). In addition, a slight growth stimulation (*p* < 0.05) was observed for nano-SiO_2_ (aq) at the lowest tested concentration (1.9 mg L^−1^). In general, there is consensus in the scientific community about the effect of NM size on organisms. Our results suggest that for the algal growth inhibition assay, there was no obvious relationship between the particle size and toxicity. For example, nano-CuO and nano-ZnO showed high particle agglomeration when suspended in MBL (Table 1) and induced high toxicity to *R. subcapitata*. However, nano-TiO_2_ showed low toxicity to microalgae despite agglomeration, while nano-SiO_2_ (aq), for which a smaller particle average size was recorded, did not display toxic effects to *R. subcapitata* up to 12.5 mg L^−1^.

It is well known that the toxicity of nano-CuO to organisms has been associated with the particles themselves [40] and/or the release of metal ions from NM [41]. In the present work, the amount of Cu^+2^ ions released from nano-CuO was very low (<0.05 mg L^−1^) and, thus, the observed toxicity may be explained by other mechanisms related to nano-CuO per se. In this context, due to the dark color of the nano-CuO suspensions and their high particle agglomeration in MBL, the attachment of nano-CuO onto the algal surface could have led to the physical shading effect, reducing the availability of light, as reported for other dark NM [42]. However, this effect alone is not enough to explain the nano-CuO toxicity to algae. The strong effect of nano-CuO on *R. subcapitata* could have been caused by the direct physical contact with algal cells followed by NM internalization, leading to deleterious effects, such as plasma membrane and cellular organelle damage. As suggested by Melegari et al. (2013), after the attachment of nano-CuO onto the algal surface, lipid peroxidation may occur, giving rise to new pores in the membrane which may contribute to nano-CuO internalization [40]. Once internalized, nano-CuO can be intracellularly transformed in nano-Cu_2_O, which is a chemical entity that releases Cu^+2^ [43], inducing high toxic effects by generating reactive oxygen species (ROS) [44]. Although the pore size of the cell wall of algae ranges from 5 to 20 nm, the size of these pores can be increased in the reproductive phase and even the interaction between the NM and algae can lead to the generation of larger pores [45]. However, in fact, all of these studies demonstrate that the mechanisms of NM interaction with cells are diverse, and all may play their role in NM toxicity.

Our results are in agreement with some studies showing that, in general, nano-ZnO is much more toxic than other metal oxide NM [46]. Although there are many studies focusing on the toxicity mechanisms of nano-ZnO on microalgae, the mechanism of toxicity of these NM remains unclear. A great deal of experimental evidence has suggested that the toxicity of nano-ZnO is mainly driven by the release of Zn^+2^ from NM [41], while other studies have shown that the release of Zn^+2^ cannot explain, by itself, the high toxicity of nano-ZnO [47]. In the present study, the nano-ZnO suspension in MBL contained nano-ZnO agglomerates and dissolved Zn^+2^ ions (Table 1). Therefore, these two factors may have contributed to inhibiting the growth of microalgae. However, it was observed that 72-h EC_50_ for nano-ZnO (4.86 mg L^−1^) was higher than the Zn^+2^ concentration dissolved in MBL (0.7 mg L^−1^), which suggests that the toxicity of nano-ZnO was not driven only by the dissolved ions. Even in an agglomeration state, nano-ZnO was able to cause severe toxicity to algae at low concentrations. Aravantinou et al. (2017) also suggested that nano-ZnO in large aggregates can be deposited on the algal surface, leading to toxic effects [48]. In this case, it is highly probable that the nano-ZnO toxicity mechanism can be similar to the one described above for nano-CuO. Inside the cells, nano-ZnO may suffer from dissolution into Zn^+2^, leading to the toxicity on microalgae, as demonstrated by other studies [49,50].

As described above, in our study, anatase nano-TiO_2_ showed low toxicity to *R. subcapitata,* as algae growth only decreased at 20 mg L^−1^ (*p* < 0.05). Since we tested anatase nano-TiO_2_ functionalized with HNO_3_, we hypothesize that this functionalization decreased the production of ROS by nano-TiO_2_ or reduced the direct interaction between NM and the microalgae cellular wall. Generally, nano-TiO_2_ anatase has been demonstrated to be more toxic than nano-TiO_2_ rutile since this type of crystal structure is more chemically reactive, resulting in an increase in ROS generation [51]. Nevertheless, Nogueira et al. (2015) also observed that anatase nano-TiO_2_ (non-functionalized) stimulated *R. subcapitata* growth at concentrations of 16 and 20 mg L^−1^ and at a high agglomeration state (average particle size: 1225 ± 26 nm) [18].

From the obtained results, both nano-SiO_2_ showed no toxicity to algae at all the tested concentrations, except for the highest (20 mg L^−1^). Hydrophilic nano-SiO_2_, which is a fumed NM, presented higher particle agglomeration in algae culture medium, while nano-SiO_2_ (30% aq. suspension), colloidal NM, showed low particle agglomeration. It is well known that the fumed nano-SiO_2_ has greater ability to produce hydroxyl radicals in comparison to colloidal nano-SiO_2_, then inducing higher toxic effects. However, if we examine our data, the type of nano-SiO_2_, as well as the particle size, were not relevant to the toxicity results, since both formulations showed similar behavior regarding their effect on *R. subcapitata.* Our data are in agreement with other studies, even testing other forms of this NM, which reported 72-h EC_20_ values of 20 ± 5 and 28.8 ± 3.2 mg L^−1^ in *R. subcapitata* for commercial colloidal silica LUDOX^®^ with hydrodynamic size of 12.5 and 27 nm, respectively [52]. Yu et al. (2018) also observed a 96-h EC_50_ = 1180.725 mg L^−1^ for commercial nano-SiO_2_ even in the agglomeration state (hydrodynamic size of 570.27 ± 27.83 nm in TAP (Tris-Acetate Phosphate medium) in *Chlamydomonas reinhardtii* [53].

### 3.3. Growth Inhibition Effect of NM on L. Minor

**Figure 2:**
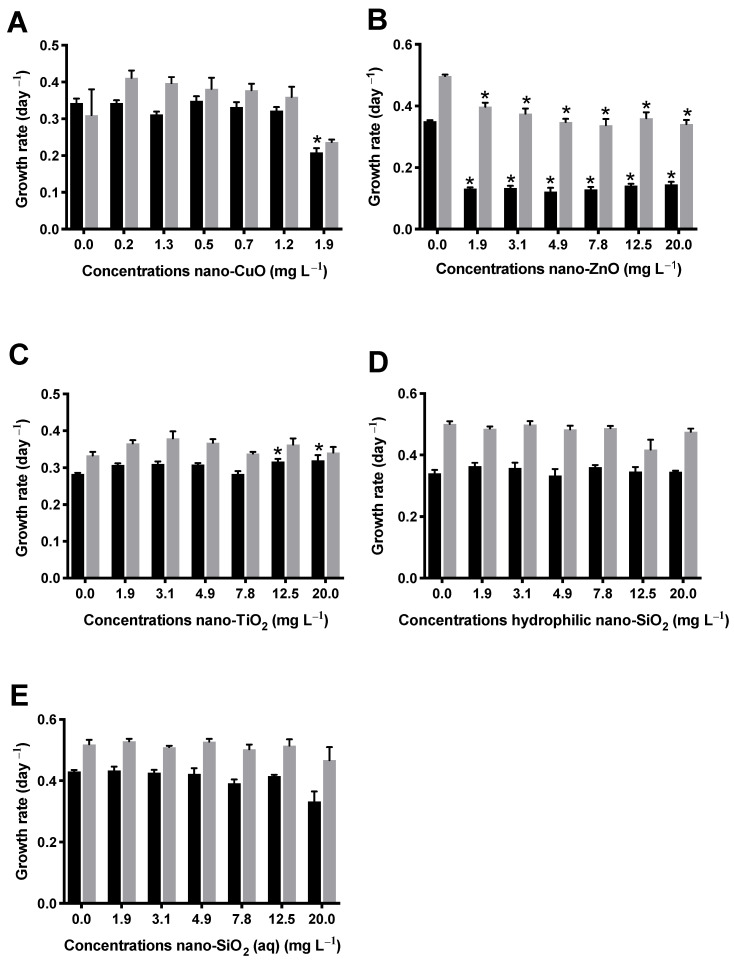
Average growth rate (day^−1^) regarding the frond number (black bars) and dry weight of fronds (grey bars) of *L. minor* exposed to the different concentrations of nano-CuO (**A**), nano-ZnO (**B**), nano-TiO_2_ (**C**), hydrophilic nano-SiO_2_ (**D**), nano-SiO_2_ (aq) (**E**) for 7 days. The experiments were conducted in triplicate and the results are shown as the mean ± standard deviation (ANOVA followed by Dunnett´s test, * *p* ˂ 0.05 compared to control).

The effects of NM on *L. minor* growth assessed by frond number and dry weight after 7 days of exposure are represented in Figure 2. Nano-CuO was the most toxic NM to *L. minor* among the other tested NM, followed by nano-ZnO. As shown in Figure 2A, after 7-day exposure, the dry weight of the plants was not significantly influenced when compared with the unexposed group. However, the number of *L. minor* fronds was drastically decreased, by 41.17%, after nano-CuO exposure at 1.9 mg L^−1^. The Cu^2+^ ions can inhibit photosystem II activities [54] and cause oxidative stress in different aquatic species, as reported by previous studies [55,56]. Yue et al. (2018) attributed the significant reduction of 32 and 33% in frond number and dry weight, respectively, in *L. minor* exposed to 0.15 mg L^−1^ of commercial nano-CuO, for 7 days, to the internalization of nano-CuO (or the Cu^+2^ ions released from NM) by roots, followed by the generation of ROS, mainly H_2_O_2_ and OH radical species [57]. On the contrary, Shi et al. (2011) observed that nano-CuO synthesized in the laboratory, per se, showed higher toxicity to the aquatic plant *Landoltia punctate* in comparison to the free Cu ions, after exposure to 1.0 mg L^−1^ nano-CuO for 9 days, which inhibited 50% of plant growth [58]. In this case, the synthesis of nano-CuO may have involved other compounds or left impurities responsible for the highest toxicity when compared to the commercial NM. Nano-ZnO (Figure 2B) was also toxic to *L. minor*, decreasing significantly the plant growth regarding both frond number and dry weight of fronds at all the tested concentrations in comparison to the control group. In the present study, DLS measurements showed the high particle agglomeration of both NM (nano-CuO and nano-ZnO) after their dispersion in Steinberg medium. In addition, the highest concentration of the dissolved ions, from both NM, was observed in *L. minor* medium. Therefore, soluble Cu^2+^ and Zn^2+^ ions may have been responsible, at least in part, for the toxic effects detected in *L. minor*, as previously described for the microalgae *R. subcapitata* (Figure 1). As shown in other reports, free zinc ions were absorbed by plants and contributed to the toxicity of commercial nano-ZnO to *Spirodela polyrhiza L.* at 50 mg L^−1^ [59]. Chen et al. (2016) also attributed the toxicity of commercial nano-ZnO to *L. minor* to the dissolved Zn ions in the medium at 10 mg L^−1^ of ZnO and at low pH [60].

For nano-TiO_2_ stabilized by HNO_3_, no growth inhibition was detected when *L. minor* plants were exposed even at the highest tested concentrations (Figure 2C). In fact, there was a slight stimulation at the highest concentrations regarding the frond number (*p* < 0.05). Nogueira et al. (2015) found no toxicity of nano-TiO_2_ anatase (non-functionalized) for *L. minor* at concentrations up to 20 mg L^−1^ after 7 days of exposure [18]. These results confirm once again that functionalization with HNO_3_ did not enhance the toxicity of nano-TiO_2_.

According to the obtained results, both nano-SiO_2_ suspensions were not toxic to *L. minor* at the tested concentrations (Figure 2D,E). To the best of our knowledge, there are no studies regarding the toxicity of nano-SiO_2_ to *Lemna* species. In fact, although Si is not an essential element for plant growth, Si can be accumulated by aquatic plants, leading to several beneficial effects, such as resistance and protection against pathogens and metals’ toxicity [61].

### 3.4. Acute Effects of NM on D. Magna

**Figure 3:**
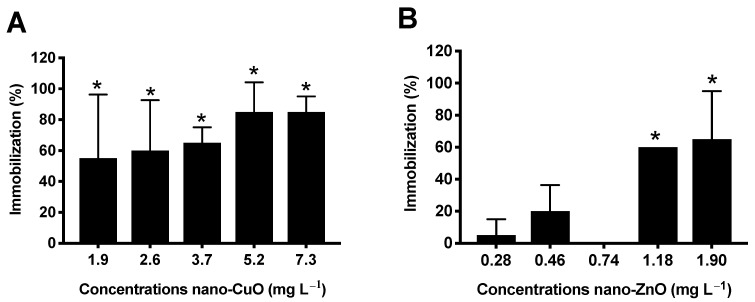
Percentage of *D. magna* immobilization exposed to nano-CuO (**A**) and nano- ZnO (**B**) suspensions for 48 h. Values are reported as the mean of four replicates ± standard deviation (ANOVA followed by Dunnett´s test, * *p* < 0.05 compared to control). Along the toxicity assays, the neonatal immobilization percentage in the control group was 0%.

The acute toxicity to *D. magna* was recorded after 24 and 48 h of exposure. *D. magna* survival was dramatically affected by both nano-CuO and nano-ZnO, showing a 48 h-EC_50_ of 1.78 mg L^−1^ for nano-CuO and 1.33 mg L^−1^ for nano-ZnO (Figure 3 and Table 2).

Nano-CuO displayed a dose-dependent response in *D. magna*, with only 15% of organisms with activity at the maximum tested concentration (7.3 mg L^−1^), after 48 h of exposure (Figure 3A). Nano-ZnO was shown to be even more toxic, since at the maximum concentration tested (1.9 mg L^−1^), only 35% of organisms with activity were recorded after 48 h of exposure (Figure 3B). As observed in the microalgae growth inhibition test, the toxicity of NM was not related with their particle size. As shown in Table 1, even in a high particle agglomeration state, nano-ZnO and nano-CuO induced high toxicity to the cladocerans, while nano-TiO_2_ and hydrophilic nano-SiO_2_ (both with high particle agglomeration) and SiO_2_ (aq) (particle size of 12.51 nm in ASTM) did not affect this filter feeding organism. Regarding the toxicity of nano-CuO on *D. magna*, it has been attributed to the presence of Cu ions in the medium. However, in our study, the concentration of dissolved ions in ASTM was 0.9 mg L^−1^ for nano-CuO (Table 1), and due to this low concentration of dissolved ions, it is highly probable that the toxicity of nano-CuO is not driven by Cu ions only. Considering that daphnids are filter feeding organisms, NM aggregates may have exerted their effect through gut clogging or through physical interaction with antennae.

Concerning nano-ZnO toxicity, the concentration of Zn ions in ASTM was of 3.4 mg L^−1^, and therefore, the toxicity of nano-ZnO to neonates can be related, at least in part, to the presence of Zn ions dissolved in the medium, as was also suggested by other studies testing commercial nano-ZnO in *D. magna* at concentrations ranging from 0.08 to 8 mg L^−1^ [62]. In addition, it is well known that *D. magna* are able to ingest and accumulate NM smaller than 50 μm [63]. Therefore, in this context, we believe that both nano-CuO and nano-ZnO could have been filtered and ingested by the daphnids, as reported by Xiao et al. (2015) [64]. These authors estimated a LC_50_ of 0.99 mg L^−1^ (nano-ZnO) and 0.093 mg L^−1^ (nano-CuO) for *D. magna* and concluded that, at concentrations of nano-CuO and nano-ZnO above 0.1 mg L^−1^ and 1.0 mg L^−1^, respectively, the particles exerted a significant effect on daphnids compared to their soluble salts due to the ingestion and adhesion effect of NM to daphnids carapace.

As seen in Table 2, the results revealed no toxicity of nano-TiO_2_ and nano-SiO_2_ (aq) to *D. magna*, resulting in 100% of active organisms even at 20 mg L^−1^. As described previously, nano-TiO_2_ anatase tested in this study was stabilized by HNO_3_, and there are no other data regarding the toxicity of this NM stabilized by NNO_3_. However, other studies testing the acute toxicity of nano-TiO_2_ anatase to daphnids indicate that this stabilization may have contributed to reducing the toxicity of these NM. For example, Novak et al. (2018) obtained an NOEC of 1 mg L^−1^ and LOEC of 10 mg L^−1^ (lower than that obtained in our study) after 48-h exposure of *D. magna* to nano-TiO_2_ in anatase form without the presence of stabilizer agents (primary particle of 5 nm and hydrodynamic size in ISO water of 375 nm) [65].

### 3.5. Critical Analysis of Available Ecotoxicological Data for Risk Assessment Purposes

As shown in Table S1 in the Supplementary Materials, which only presents published studies that followed standard protocols to test the toxicity of the NM (ISO or OECD guidelines), it is evident that different works tested different forms of the same NM (different physicochemical properties, surface modifications, functionalization of NM), the number of test species is limited, the characterization of the NM is frequently performed in a water suspension and not in the test media, and all these aspects contribute to the lack of useful data for the derivation of environmental threshold levels, by probabilistic methods—for example, through the application of species sensitivity distributions [66]. This is unfortunately true for all the NM under evaluation in this study, despite being apparently the most studied NM—for example, nano-TiO_2_. Regarding nano-CuO, a direct comparison of acute toxicity to *D. magna* was only possible between our study and the studies of Adam et al. (2015) [67], Kim et al. (2017) [68] and Muna et al. (2019) [69], for NM primary sizes between 20 and 50 nm (Table S1). EC_50_ values for *D. magna* acute immobilization tests were 12 times higher [70] than those found in our studies, probably due to the use of a different test medium. The same occurred when the data reported by Aruoja et al. 2009 [41], for *L. minor* growth inhibition, are compared with ours. Once again, and despite the same test protocol being followed, the used test media were different, and the primary size of particles reported by these authors is smaller than that reported in our study. No toxicity endpoints for other test species can be obtained from the literature for nano-CuO, except for *H. incongruens* [69], if we consider the above range of NM sizes, rather than looking to a specific particle size. This may be considered acceptable if we take into account that it is difficult to expose organisms to NM at the particle size reported by the manufacturer, since particle aglomeration frequently occurs in the test media. Table 3 displays the toxicity endpoints that were used for the estimation of a PNEC value for nano-CuO. Values expressed in concentration of Cu were converted to concentration of CuO. Whenever available, only chronic endpoints were taken into account and an assessment factor of 10 was used, considering that three endpoints for three species from different throphic levels were available. The PNEC value obtained is 2.65 times higher than that obtained by Chen et al. (2018) (0.049 mg L^−1^) [33], which is in part unexpected, since the assessment factors are usually a more conservative aproach. Nevethless, it is not clear which data were actually used by these authors.

**Figure 4:**
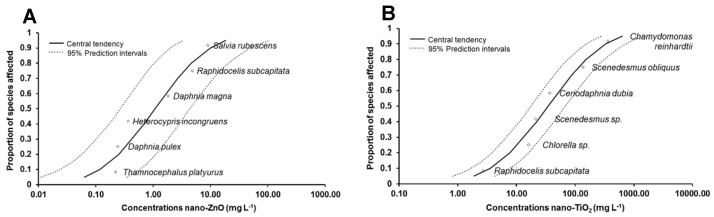
Species sensitivity distribution curves obtained by fitting toxicity endpoints to linearized log-normal model to toxicity endpoints available for nano-ZnO (**A**) and nano-TiO_2_ (**B**).

For nano-ZnO, more toxicity data are available compared with the other NM (Table S2), some of which helped to confirm the sensitivity of *D. magna*, since they provided EC_50_ values of the same order of magnitude, if we consider particle sizes > 100 nm. The exception is the study of Zhu et al. (2009), which showed a level of sensitivity two-times lower than that recorded in our study, for a NM wth a similar primary size [78]. Further, some additional species were tested, including, for example, *H. incongruens*, *D. pulex*, *S. rubens* and *T. platyurus* [48,69,72,73]. For these NM, it was possible to generate an SSD as data for six different species (Figure 4A). However, to overcome the lack of data, it was necessary to combine the data from acute and chronic tests and for a range of primary sizes, as shown in Table 3. The HC_5_ recorded was once again three-times greater than that proposed by Chen et al. (2018). However, as mentioned above, it is not clear which data were used by these authors to generate HC_5_ [33].

Regarding nano-TiO_2_ (Table S3), considered the most studied metal oxide NM, the diversity of the NM tested is in fact very high, making it difficult to obtain sufficient data for the derivation of HC_5_ values for each one, using probabilistic methodologies. However, it is clear that our study is the only one providing toxicity data for hydrophilic TiO_2_ stabilized by HNO_3_. The data selected allowed us to estimate an HC_5_ for TiO_2_ anatase of 1.89 mg L^−1^ (primary particle size < 25 nm, anatase), which was different from those proposed by Chen et al. (2018) [33] (Figure 4B). In our study, we provided a threshold only for anatase nano-TiO_2_. However, we have combined acute and chronic endpoints, since otherwise, the available data were even fewer. Chen et al. (2018) did not take into account the difference in the mineral nature of this NM. Regarding the NM tested in our study (nano-TiO_2_ stabilized with HNO_3_, anatase), only one endpoint was used to estimate a PNEC value, thus providing an extremely protective value, which points towards the higher risk of this NM when compared with their non-stabilized form. This value is not in agreement with the lowest toxicity suggested for this NM and by the LOEC and NOEC values recorded. The same occurred for nano-SiO_2_ (Table S4), which is the less studied NM, with very few data available supported by standard protocols and thus useful for risk assessment purposes. The lack of data available gave rise to two PNEC values, for the silica-based NM tested in this study, which are also extremely protective considering that almost all the ecotoxicological data point towards the low toxicity of these NM.

### 3.6. Cytotoxicity of NM to Human Cell Lines

**Figure 5:**
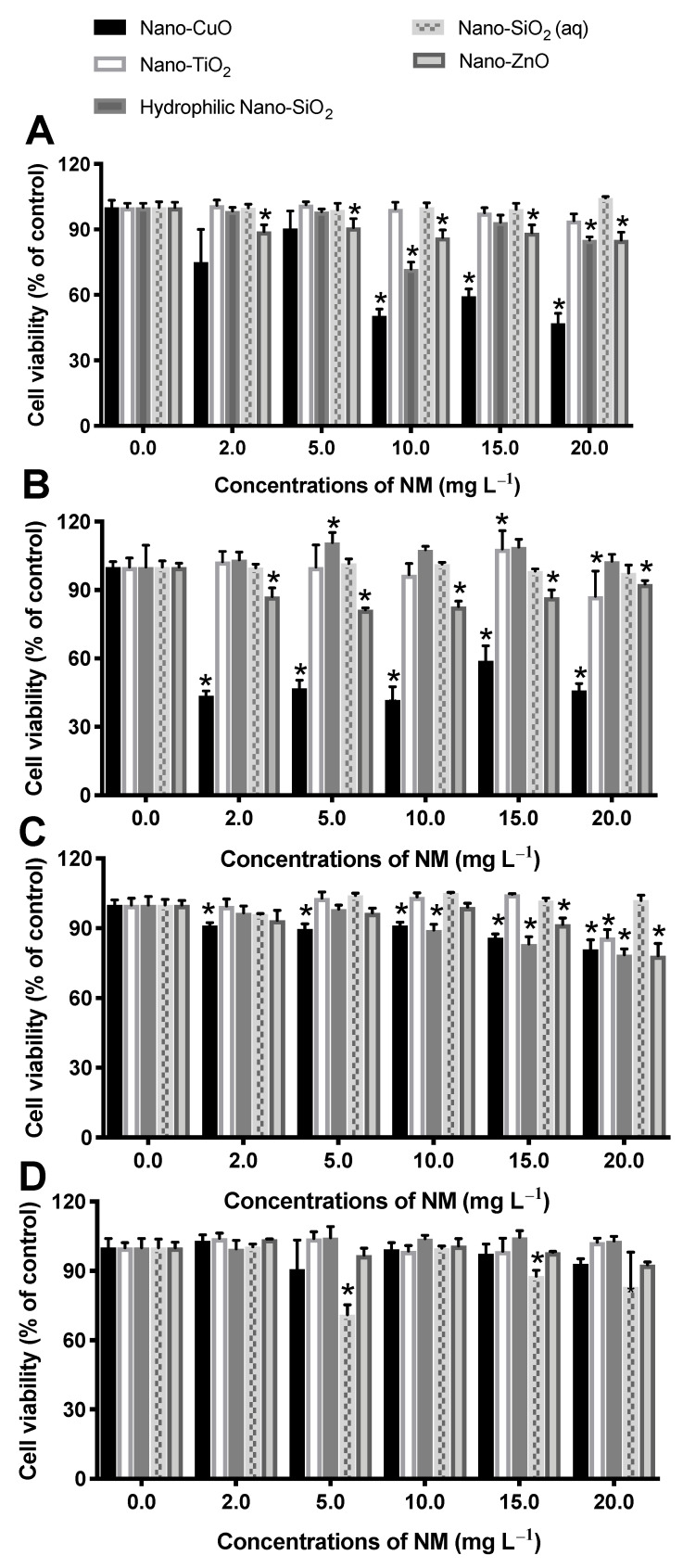
Cell viability of Caco-2 (**A**), HepG2 (**B**), SV-80 (**C**) and HaCaT (**D**) cells, after 48 h exposure to different NMs, as denoted, at concentrations ranging from 2 to 20 mg L^−1^. Results are expressed as the percentage of control, and for each cell line, each concentration was tested in quadruplicate. Control group corresponds to non-exposed cells, set as 100% of cell viability. Significant differences in cell viability compared to the control group are indicated by * *p* < 0.05 (ANOVA followed by Dunnett’s test).

Since in vitro cytotoxicity studies may elucidate the mechanisms of toxicity and evaluate the acute toxicity of the compounds, in this study, Caco-2, HepG2, HaCaT and SV-80 cell lines were used to assess the impact of NM exposure on humans, through different pathways. Figure 5 shows the cell viability data for the different cell lines incubated with NM, for 48 h, at concentrations ranging from 2 to 20 mg L^−1^. According to the results, nano-CuO was the most toxic NM to intestine and liver cells (Figure 5A,B). Nano-CuO caused a significant reduction in Caco-2 cell viability (~40–50%) at concentrations ≥ 10 mg L^−1^ after 48 h of exposure (Figure 5A). For HepG2 cells, nano-CuO was able to induce higher toxicity even at the lowest tested concentration (2 mg L^−1^), reaching a plateau (cell viability around 45%; Figure 5B). However, these NM showed low toxicity to the lung and skin cell lines (Figure 5C,D). There are very few studies that investigate the effect of nano-CuO on human cell lines and how nano-CuO could induce cytotoxicity is still unclear. Previous studies have demonstrated that the release of Cu^+2^ ions from nano-CuO may be responsible for the high toxicity of these NM [79]. Therefore, since, in our study, nano-CuO exhibited some solubility in the cell culture medium (4.3 mg L^−1^), it is possible that free Cu ions also contributed to the nano-CuO effects. In the same way, Piret et al. (2012) showed that at 25 mg L^−1^, spherical and rod-shaped commercial nano-CuO (average size of 50 and 12 nm) decreased Caco-2 cell viability in 20 and 30%, respectively, after 48 h of exposure. These authors also attributed the observed toxicity to the release of Cu^+2^ and to the shape of NM [80]. The same authors reported a decrease in HepG2 cell viability after exposure to commercial nano-CuO at concentrations of 20–80 mg L^−1^ of Cu, attributing this effect to the production of ROS [81]. As described above, nano-CuO did not induce any toxic effect on HaCaT cells (Figure 5D), maintaining the cell viability above 70% when compared to the control group. Similar results were obtained by Luo et al. (2014), who reported low toxicity of synthesized nano-CuO (5–7 nm) in HaCaT human keratinocytes at 20 mg L^−1^ after 24 h of exposure [82].

Regarding the effects of nano-CuO on SV-80 cells, a decrease of 20% in cell viability at 20 mg L^−1^ was observed. There are no studies regarding the toxicity of nano-CuO on the SV-80 cell line. However, for comparison, other studies have reported a decrease in the viability of human lung epithelial cells (A549 cell line) in ~90 and 93% after 18 h of exposure to 40 and 80 mg L^−1^ of commercial nano-CuO (hydrodynamic particle size of 220 nm), respectively [83].

In the present study, nano-ZnO was shown to be biocompatible for all selected human cell lines at the tested concentrations. The decrease in cell viability was more pronounced for SV-80 cells (21.8%) after exposure to nano-ZnO at 20 mg L^−1^. The data regarding the in vitro toxicity of nano-ZnO to human cells are very controversial. Abbot et al. (2013) only detect high toxicity (50% reduction in cell viability) for commercial nano-ZnO (hydrodynamic particle size 212–260 nm) to Caco-2 cells, after 48 h of exposure at 100 mg L^−1^ [84]. However, Abbasi-Oshaghi et al. (2016) demonstrated a significant decrease in Caco-2 cell viability (40%) at 20 mg L^−1^, after 24 h of exposure to synthesized nano-ZnO in the laboratory (20–30 nm measured by TEM) [85]. Sharma et al. (2012) also showed that exposure to 14–20 mg L^−1^ of commercial nano-ZnO (hydrodynamic particle size of 267 nm) for 12 h reduced the HepG2 cells’ viability and induced cell death by apoptosis [86]. As seen in Figure 5, nano-ZnO also did not induce any toxic effects on human keratinocyte cells (HaCaT). Vinardell et al. (2017) showed an IC_50_ of 50 mg L^−1^ after 48 h of HaCaT cells’ exposure to commercial nano-ZnO with 100 nm [87], which is in agreement with our study that reports no toxicity at concentrations up to 20 mg L^−1^. Lee et al. (2012) also observed a low effect of commercial nano-ZnO at 20 mg L^−1^ (decrease in HaCaT cell viability of 25% compared with the control cells) after 24 h of exposure [88]. The in vitro cell toxicity of nano-ZnO is generally attributed to the release of Zn^+2^ from nanoparticles [89] as well as to the induction of cellular oxidative stress due to the generation of ROS [89,90,91]. However, the toxicity mechanisms of nano-ZnO are not clearly established. A study performed by Chen et al. (2019) showed that at concentrations above 16 mg L^−1^, the toxicity of commercial nano-ZnO (hydrodynamic particle size 37–200 nm) to HepG2 cells was ascribed to the release of Zn^+2^ after 24 h of exposure [16]. However, in our study, the concentration of Zn ions in DMEM was very low (1.7 mg L^−1^) and, therefore, it is possible that the absence of toxicity can be attributed to the low concentration of soluble ions in the cell culture medium.

From our study, nano-TiO_2_ anatase was not toxic to any of the cell lines at the tested concentrations, which is in agreement with the previously reported data. As demonstrated by several works, data for the cytotoxicity of nano-TiO_2_ are consistent despite the various types of TiO_2_ NM tested (different crystalline structures, particle size, surface charge), cell culture medium used, time of exposure and methods followed to evaluate the cell viability.

Commercial nano-TiO_2_, even in the particle agglomeration state (hydrodynamic particle size of 212–260 nm in Caco-2 cell medium), was not toxic to Caco-2 cells at concentrations ranging from 0.1 to 100 mg L^−1^ after 48 h of exposure [84]. Tada-Oikawa et al. (2016) observed no toxicity to Caco-2 cells after 24-h exposure to commercial nano-TiO_2_ with different crystal structures (anatase and rutile) and particle sizes (primary particle sizes of 50 and 100 nm; hydrodynamic particle sizes of 227.78 and 253.4 nm in DMEM containing serum) [92]. Wang et al. (2011) also did not observe toxic effects for nano-TiO_2_ (crystal structure not reported; particle size of 17–64 nm provided by TEM) in HepG2 cells after 48-h exposure at concentrations ranging from 25 to 200 mg L^−1^ [93]. Rutile nano-TiO_2_ (hydrodynamic particle size of 447.2 nm in HepG2 serum-free medium) was not toxic to Caco-2 cells at concentrations up to 25 mg L^−1^, after 24 h of exposure [94]. In fact, some studies postulate that the presence of serum in the cell culture medium can led to a reduction in particle agglomeration [95]. In our study, the cytotoxicity assays were conducted in FBS-free DMEM, resulting in high particle agglomeration. Nevertheless, given all the information available, and despite the nano-TiO_2_ tested in our study to be different from the other studies, since it was stabilized with HNO_3_, the results conclusively point towards the biocompatibility of these NM.

No sign of severe toxic effects, in all tested cell lines, was observed for both nano-SiO_2_ suspensions in DMEM after 48 h of incubation. However, as described previously for the other NM, there are several types of nano-SiO_2_ (crystalline, amorphous, mesoporous) synthesized and tested by different methodologies and exposure time, respectively, which in turn make it difficult to compare their toxicological effects [96]. In our study, we tested the commercial fumed amorphous nano-SiO_2_ with hydrophilic properties (probably due to a surface functionalization which was not specified by the manufacturer; hydrodynamic particle size of 162.86 nm in DMEM) and the commercial colloidal amorphous nano-SiO_2_ in aqueous suspensions (hydrodynamic particle size of 119.56 nm in DMEM) after 48 h of exposure through the AlamarBlue (AB) method. In opposition to our results, Ahmad et al. (2012) showed that commercial amorphous nano-SiO_2_ (hydrodynamic particle size of 96 nm in cell culture medium) at concentrations up to 10 mg L^−1^ did not decrease significantly HepG2 cell viability measured by MTT and neutral red uptake (NRU) methods and for a longer time period of exposure (72 h) [97]. Using commercial amorphous nano-SiO_2_ (primary particle size of 15 nm) and the MTT cell viability method, for a shorter period of time (24 h), Gong et al. (2017) observed a decrease of almost 50% in HaCaT cell viability at 20 mg L^−1^ [98], which was the highest concentration tested in our study (Figure 5D). Contrarily, Liang et al. (2014) observed no toxicity for HaCaT cells when exposed to nano-SiO_2_ (particle size of 50 nm measured by TEM) synthesized by the Stöber method at 25 mg L^−1^ after 4 h of exposure [99]. Regarding HepG2 and Caco-2 cells, Andreani et al. (2014) also evaluate the cytotoxicity of synthesized colloidal amorphous nano-SiO_2_ (lyophilized using trehalose 10% (w/v, 1:1) as cryoprotectant; hydrodynamic particle size of 290 nm measured in ultrapure water) by the Stöber sol–gel method and observed no toxicity for Caco-2 and HepG2 cells at concentrations of 0–500 mg L^−1^ after 48 h of exposure measured by AB assay [30]. Different cytotoxicity results have been obtained by Li et al. (2011), who compared four types of synthesized colloidal nano-SiO_2_ with different sizes (hydrodynamic particle sizes of 628.5, 127.3, 108.8 and 82.2 nm measured in DMEM after 24 h) and demonstrated size-dependent cytotoxicity of nano-SiO_2_. However, only nano-SiO_2_ with smaller particle size decreased the viability of HepG2 cells (the cell viability was determined by the WST-8 cell counting kit (CCK-8)) in ~20 and 30% at a concentration of 12.5 and 25 mg L^−1^, respectively, after 24 h of exposure. The other nano-SiO_2_ did not significantly reduce the cell viability at concentrations up to 100 mg L^−1^ [100].

As described above, cytotoxicity data regarding the metal oxide NM (commercial or lab synthesized) may differ depending on the type of NM, cells involved, toxicological assays and incubation time. Gastrointestinal and respiratory tracts, as well as the skin, are the organs that can be widely exposed to the toxic effects of these entities and, therefore, more research and the harmonization of experimental processes by standard protocols are urgently needed to guarantee the safe application of NM [101]. In addition, the lack of consideration of a NM characteristics–toxicity relationship in most toxicological studies can lead to uncertain or erroneous conclusions [101].

## 4. Conclusions

In the present study, the effect of different metal oxide NM, namely nano-CuO, nano-TiO_2_, nano-SiO_2_ and nano-ZnO, on three aquatic freshwater species (*R. subcapitata*, *D. magna* and *L. minor*) and on human cell lines (Caco-2, HepG2, SV-80 and HaCaT) has been evaluated. From the data obtained in this study, most of the NM showed the tendency to agglomerate after their dispersion into the different culture media used for the maintenance of distinct test species. However, the particle agglomeration did not prevent the NM from exerting their toxic effects. Nano-CuO and nano-ZnO, despite the particle agglomeration, were consistently toxic to freshwater organisms, *R. subcapitata*, *D. magna* and *L. minor*, at the tested concentrations. The opposite results were recorded for nano-TiO_2_ (high particle agglomeration state) and nano-SiO_2_ (low particle agglomeration for nano-SiO_2_ (aq) and high particle agglomeration for hydrophilic nano-SiO_2_), in which no toxicity was observed for the tested aquatic species.

Concerning the NM effect on human cell lines, results showed that the viability of SV-80 and HaCaT cells was little affected by NM after 48-h incubation, with the cell viability remaining 70% higher than the control. However, nano-CuO was very toxic to Caco-2 and HepG2 cells, especially at the highest tested concentrations, while nano-TiO_2_, nano-SiO_2_ and ZnO were not cytotoxic to the different human cell lines. No relationship was found between NM particle size and cytotoxicity for the tested NM. The toxicity can be related to the adsorption of NM to biological membranes or to the generation of ROS, but also to the presence of the ions in the cell medium, mainly for nano-CuO and nano-ZnO suspensions.

An extensive bibliographic search and compilation of data available in the literature demonstrated that data for the ecotoxicity and cytotoxicity of NM, even for those NM apparently more tested, are still very scarce, since most of the published studies were performed using different forms of NM. The risk thresholds estimated (HC_5_ and PNEC values) are still highly protective, since they are based on few toxicity data. However, this study provides consistent evidence of the potential risks of both nano-CuO and nano-ZnO to aquatic organisms and also their effect on public health.

Although great effort has been made by OECD and ISO to adapt the standard protocols to the specificities of nanomaterials, there are no new standard protocols to replace those used in this study or in the previous studies reviewed in this manuscript. For example, for aquatic tests, OECD published in 2020 a guidance document on the aquatic and sediment toxicological testing of nanomaterials (Guidance Document 317) that is not intended to replace existing protocols but to adapt the existing Test Guidelines for reliable NM testing. Therefore, the harmonization of experimental processes by standard protocols and follow-up of OECD guidelines already published, such as OECD 318 (2017), must be considered and respected in order to guarantee data quality as well as their application in risk assessment evaluation.

## Figures and Tables

**Table 1:** Average hydrodynamic size ± standard deviation (SD), polydispersity index (PI), average zeta potential (ZP ± SD) and concentration of the free metal ions recorded in the nanomaterials (NM) dispersions prepared in different culture media at 20 mg L^−1^.

NM	Culture Medium	Hydrodynamic Particle Size (nm ± SD)	PI	ZP (mV ± SD)	Free Metal Ions (mg L^−1^)
**Nano-CuO**	MBL	223.3 ± 76.3	0.34	−22.8 ± 2.9	<0.05
	ASTM	>1 µm	0.76	−5.9 ± 0.3	0.9
	Steinberg	>1 µm	–*	−7.9 ± 1.1	8.5
	DMEM without FBS	>1 µm	–*	−2.3 ± 0.7	4.3
**Nano-TiO_2_**	MBL	770.5 ± 174.8	0.92	−21.6 ± 0.8	0.15
	ASTM	>1 µm	–*	−9.4 ± 2.7	0.65
	Steinberg	899.7 ± 75.9	0.92	−9.9 ± 0.4	3.9
	DMEM without FBS	>1 µm	–*	−4.3 ± 0.7	0.14
**Hydrophilic nano-SiO_2_**	MBL	209.2 ± 180.0	0.86	−27.3 ± 3.8	–
	ASTM	235.2 ± 104.2	0.44	−15.3 ± 1.0	–
	Steinberg	195.5 ± 85.6	0.43	−8.4 ± 1.8	–
	DMEM without FBS	162.9 ± 147.6	0.90	−21.3 ±1.4	–
**Nano-SiO_2_ (aq)**	MBL	11.8 ± 2.6	0.22	−25.7 ± 2.0	–
	ASTM	12.5 ± 1.6	0.13	−13.9 ± 1.3	–
	Steinberg	50.0 ± 26.0	0.52	−9.8 ± 0.6	–
	DMEM without FBS	119.6 ± 57.0	0.47	−10.8 ± 6.4	–
**Nano-ZnO**	MBL	178.6 ± 116.6	0.65	−27.5 ± 1.1	0.7
	ASTM	281.8 ± 214.8	0.76	−2.2 ± 0.1	3.4
	Steinberg	>1 µm	–*	−10.2 ± 1.2	5.1
	DMEM without FBS	300.1 ± 195.2	0.65	−6.7 ± 0.1	1.7

Note: * Indicates that DLS measurements were not able to calculate the PI values, since the cumulant method is not suitable for the highly polydispersed formulations.

**Table 2:** Effective concentrations (EC_20_ and EC_50_ values), LOEC and NOEC of NM obtained for microalgae growth inhibition and (EC_50_) for *D. magna* immobilization after 24 and 48 h of exposure.

NM	EC_20_ and EC_50_ Values for Algae Growth Inhibition (mg L^−1^)				EC_50_ Values for *D. magna* Immobilization (mg L^−1^)	
	EC_20_	EC_50_	LOEC	NOEC	24 h	48 h
Nano-CuO	3.08 (1.24–4.92)	12.77 (8.84–16.70)	3.1	1.9	5.00 (4.27–6.20)	1.78 (0.21–2.66)
Nano-ZnO	4.11 (4.00–4.21)	4.86 (4.81–4.91)	3.6	2.5	11.88 (ND)	1.33 (ND)
Nano-TiO_2_	>20	>20	20	12.5	>20	>20
Nano-SiO_2_	>20	>20	7.8	4.9	>20	>20
Nano-SiO_2_ (aq)	>20	>20	1.9	-	>20	>20

**Table 3:** Predicted no-effect concentrations (PNEC) or hazard concentrations for 5% of the species (HC_5_) and corresponding 95% confidence intervals, estimated for the NM under evaluation in this study.

	Species	Endpoint	mg L^−1^	
Nano-CuO				
Primary particle average size (20–50 nm)	*R. subcapitata*	72 h-EC_50_ (growth inhibition)	12.8	This study
	*H. incongruens*	6d-EC_50_ (mortality)	2.4	[69]
	*D.magna*	Average number of neonates	1.3	[67]
		PNEC (AF = 10)	**0.13**	
Nano-ZnO				
Primary particle average size (<100 nm)	*R. subcapitata*	72 h-EC_50_ (growth inhibition)	4.86	This study
	*D. magna*	EC_50_ (immobilization)	1.3	This study
			1.9	[69]
			3.1	[71]
			1.4	[68]
		Geometric mean		
	*H. incongruens*	6d-EC_50_ (mortality)	0.37	[69]
	*D. pulex*	48 h-EC_50_ (immobilization)	0.24	[72]
	*S. rubescens*	28d-IC_50_ (Growth rate)	8.93	[48]
	*T. platyurus*	6d-EC_50_ mortality	0.22	[73]
		HC_5_ r^2^ = 0.898	0.063 (95%CI:0.01–0.35)	
Nano-TiO_2_ anatase stabilized with HNO_3_				
Primary particle average size (4–8 nm)—anatase	*R. subcapitata*	72 h-EC_50_ (growth inhibition)	20.0	This study
		PNEC (AF = 100)	0.2	
Nano-TiO_2_ anatase				
Primary particle average (<25 nm)—anatase	*C. reinhardtii*	96 h-EC_50_ Growth rate	359.8	[53]
	*S. obliquus*	72 h-EC_50_ (growth rate)	136.9	[74]
	*Scenedesmus sp.*	72 h-EC_50_ (growth rate)	21.2	[75]
	*Chlorella sp.*	72 h-EC_50_ (growth rate)	16.12	[75]
	*C. dubia*	48 h-LC_50_ immobilization	37.04	[76]
	*R. subcapitata*	72 h-EC50 Growth rate	2.7	[77]
		HC_5_ r^2^ = 0.977	1.89 (95% CI: 0.83–4.31)	
**Nano-SiO_2_ hydrophilic**				
Primary particle average size (<25 nm)	*R. subcapitata*	72 h-EC_50_ Growth rate	7.8	This study
		PNEC (AF = 100)	0.08	
Nano-SiO_2_ (aqueous)	*R. subcapitata*	72 h-EC_50_ Growth rate	1.9	This study
Primary particle average size (10 nm)				
		PNEC (AF = 100)	0.02	
Nano-SiO_2_				
Primary particle average size (10–20 nm)	*C. reinhardtii*	72 h-EC_50_ Growth rate	1180.7	[53]
		PNEC (AF = 100)	11.8	

Note: LOECs and NOECs were not used for PNEC and HC_5_ estimations.

## Data Availability

All data have been illustrated in the manuscript.

## Supplementary Materials

The following are available online at https://www.mdpi.com/2079-4991/11/1/66/s1, Table S1: Physicochemical characteristics and toxic effects of different nano-CuO on freshwater species. The primary particle size was supported by the manufacturer. NR: data not reported; Table S2: Physicochemical characteristics and toxic effects of different nano-ZnO on freshwater species. The primary particle size was supported by the manufacturer. NR: data not reported; Table S3: Physicochemical characteristics and toxic effects of different nano-TiO_2_ on freshwater species. The primary particle size was supported by the manufacturer. NR: data not reported; Table S4: Physicochemical characteristics and toxic effects of different nano-SiO_2_ on freshwater species. The primary particle size was supported by the manufacturer. NR: data not reported.
