# Supplementary material for: Ecotoxicity to Freshwater Organisms and Cytotoxicity of Nanomaterials: Are We Generating Sufficient Data for Their Risk Assessment?
Source: Nanomaterials (Basel). 2020 Dec 30;11(1):66. doi: 10.3390/nano11010066 (PMC7824120; doi:10.3390/nano11010066)
Supplement: Supplementary file 1 [file nanomaterials-11-00066-s001.pdf]

nanomaterials

Supplementary Materials

Ecotoxicity to Freshwater Organisms and Cytotoxicity of Nanomaterials: Are We Generating Sufficient Data for Their Risk Assessment?

Tatiana Andreani [1,2,3,*], Verónica Nogueira [4], Ana Gavina [3], Saul Fernandes [3], José Luís Rodrigues [5], Vera V. Pinto [5], Maria José Ferreira [5], Amélia M. Silva [2,6], Carlos M. Pereira [1] and Ruth Pereira [3,*]

1 Centro de Investigação em Química da Universidade do Porto, CIQUP & Department of Chemistry and Biochemistry, Faculty of Sciences of the University of Porto, Rua do Campo Alegre, 4169-007 Porto, Portugal; cmpereir@fc.up.pt
2 CITAB—Centre for Research and Technology of Agro-Environmental and Biological Sciences, University of Trás-os-Montes e Alto Douro, UTAD, 5000-801 Vila Real, Portugal, amsilva@utad.pt
3 GreenUPorto—Sustainable Agrifood Production Research Centre & Department of Biology, Faculty of Sciences of the University of Porto, Rua do Campo Alegre s/n, 4169-007 Porto, Portugal; ana.gavina@fc.up.pt (A.G.); saulsimao@gmail.com (S.F.)
4 Interdisciplinary Centre of Marine and Environmental Research (CIIMAR), University of Porto, Terminal de Cruzeiros do Porto de Leixões, Av. General Norton de Matos s/n, 4450-208 Matosinhos, Portugal, veronica.nogueira@fc.up.pt
5 Centro Tecnológico do Calçado de Portugal, Rua de Fundões—Devesa Velha, 3700-121 São João Madeira, Portugal; jose.rodrigues@ctcp.pt (J.L.R.); Vera.pinto@ctcp.pt (V.V.P.); MJose.Ferreira@ctcp.pt (M.J.F.)
6 Department of Biology and Environment, University of Trás-os-Montes e Alto Douro, UTAD, Quinta de Prados, P-5000-801 Vila Real, Portugal
* Correspondence: tatiana.andreani@fc.up.pt (T.A.); ruth.pereira@fc.up.pt (R.P.); Tel.: +351-220-402-000

Table S1. Physicochemical characteristics and toxic effects of different nano-CuO on freshwater species. The primary particle size was supported by the manufacturer. NR: data not reported.

NM	Physicochemical Characterization	Origin	Freshwater species	Standard Test Protocol	Range concentrations tested, testing medium and dilution factor (DF)	Parameters	Endpoints - ECx (95% confidence intervals when available), NOEC and LOEC	References
CuO	~ 40 nm primary size; Hydrodynamic size, PI and ZP, respectively, measured at 20 mg L^{-1} in MBL medium: 223.34 ± 73.34 nm, 0.34 and, – 22.8 ± 2.95 mV	PlasmaChem	*R. subcapitata*	OECD 201	Concentrations tested: 1.9-20 mg L^{-1}<br>Woods Hole MBL medium<br>DF: 1.6	Growth rate	72-h EC_{50} = 12.77 (8.84-16.70) mg CuO L^{-1}<br>LOEC = 3.1 mg L^{-1}	Present work
CuO	~ 40 nm primary size; Hydrodynamic size, PI and ZP, respectively, measured at 20 mg L^{-1} in ASTM medium: > 1000 nm, 0.76 and – 5.90± 0.36 mV	PlasmaChem	*D. magna*	OECD 202	Concentrations tested: 1.9-7.3 mg L^{-1}<br>ASTM medium<br>DF: 1.4	Immobilization	48-h LC_{50} = 1.78 (0.21-2.66) mg CuO L^{-1}	Present work
CuO	~ 40 nm primary size; Hydrodynamic size, PI and ZP, respectively, measured at 20 mg L^{-1} in Steinberg medium: >	PlasmaChem	*L. minor*	OECD 221	Concentrations tested: 0.2-1.9 mg L^{-1}<br>Steinberg medium<br>DF: 1.6	Growth rate (frond number)	7-days LOEC = 1.9 mg CuO L^{-1}	Present work

	1000 nm, 1.00 and – 7.91± 1.10 mV							
CuO	22-25 nm primary size; Hydrodynamic size, PI and ZP, respectively, measured at 10 mg L^{-1} in AFW medium after 48h: 1826 ± 346 nm, 0.78 ± 0.023 and – 4.60 ± 0.59 mV	Intrinsiq Materials	*D. magna*	OECD 202	Concentrations tested: NR<br><br>OECD artificial freshwater (AFW)	Immobilization	48-h EC_{50} = 1.6 (1.1-3.4) mg Cu L^{-1}	[1]
CuO	22-25 nm primary size; Hydrodynamic size, PI and ZP, respectively, measured at 10 mg L^{-1} in Lake Raku waters after 48h: 393.0 ± 8.9 nm, 0.28 ± 0.026, – 18.0 ± 0.17 mV	Intrinsiq Materials	*D. magna*	OECD 202	Concentrations tested: NR<br><br>Lake Raku water	Immobilization	48-h EC_{50} = 6.3 (3.9-13) mg Cu L^{-1}	[1]
CuO	22-25 nm primary size; Hydrodynamic size, PI and ZP, respectively, measured at 10 mg L^{-1} in Lake Ülemiste waters after 48h: 440 ± 20 nm, 0.26 ± 0.018 and – 17 ± 0.46 mV	Intrinsiq Materials	*D. magna*	OECD 202	Concentrations tested: NR<br><br>Lake Ülemiste water	Immobilization	48-h EC_{50} = 28 (18-53) mg Cu L^{-1}	[1]

CuO	22-25 nm primary size; Hydrodynamic size, PI and ZP, respectively, measured at 10 mg L^{-1} MHW medium after 6 days: 2645 ± 841 nm, 1.0 ± 0.0 and – 13.0 ± 4.0 mV	Intrinsiq Materials	*Heterocypris incongruens*	OSTRAC ODTOX KIT F (based on ISO 14371)	Concentrations tested: NR<br>US EPA artificial freshwater with microalgae addition (MHW)	Mortality	6-days LC_{50} = 1.1 (1.1-1.6) mg Cu L^{-1}	[1]
CuO	22-25 nm primary size; Hydrodynamic size, PI and ZP, respectively, measured at 10 mg L^{-1} in Lake Raku waters after 6 days: 449 ± 18 nm, 0.35 ± 0.017 and – 17.00 ± 0.36	Intrinsiq Materials	*Heterocypris incongruens*	OSTRAC ODTOX KIT F (based on ISO 14371)	Concentrations tested: NR<br>Lake Raku water with microalgae addition	Mortality	6-days LC_{50} = 1.9 (1.4-3.3) mg Cu L^{-1}	[1]
CuO	22-25 nm primary size; Hydrodynamic size, PI and ZP, respectively, measured at 10 mg L^{-1} in Lake Ülemiste waters after 6 days: 452 ± 65 nm, 0.35 ± 0.0065 and – 17.00 ± 0.55	Intrinsiq Materials	*Heterocypris incongruens*	OSTRAC ODTOX KIT F (based on ISO 14371)	Concentrations tested: NR<br>Lake Ülemiste water with microalgae addition	Mortality	6-days LC_{50} = 2.20 (0.77-4.20) mg Cu L^{-1}	[1]
CuO functionalized	Hydrodynamic size, PI and ZP, respectively: 272.9 ± 14.0 nm, 0.34 ±	Synthesized	*Ceriodaphnia silvestrii*	ABTN (NBR12713)	Concentrations tested: 0.0, 7.0, 10.0, 13.0, 16.0 and 19.0 µg Cu L^{-1}	Immobilization	48-h EC_{50} = 12.6 ± 0.7 µg Cu L^{-1}	[2]

with sodium citrate	0.08 and −20 ± 14 mV, respectively				Reconstituted water (pH 7.0–7.6, conductivity 160 µS cm^{-1} and hardness 40–48 mg $CaCO_3$ L^{-1})			
CuO	< 50 nm primary size	Sigma-Aldrich	*L. minor*	ISO/CD 20079	Concentrations tested: 0.1-1000 µM<br>Steinberg medium<br>DF: 10.0	Growth rate (frond number)	7-days LOEC = 0.1µM	[3]
CuO	30-50 nm primary size; Hydrodynamic size of 400-700 nm at 10 mg L^{-1}	Alfa Aesar	*D. magna*	OECD 202	Maximum concentration tested: 10 mg L^{-1}<br>Moderately hard water (MHW)	Immobilization	48-h EC_{50} =1.0 ± 0.3 mg CuO L^{-1}	[4]
CuO	< 50 nm primary size; Hydrodynamic size and PI respectively, measured at 1.438 mg L^{-1} in ultrapure water: 312 nm (267-364 nm) and 0.5	Sigma-Aldrich	*D. magna*	OECD 211	Concentrations tested: 0.037-1.438 mg Cu L^{-1}<br>Concentration expressed in terms of metal and not metal oxide content<br>OECD recommended ISO test medium<br>DF: 2.50	Average number of neonates	21-days EC_{50} = 1.0 mg Cu L^{-1} (0.7-1.45)	[5]

CuO	30-40 nm primary size; Hydrodynamic size and ZP, respectively, measured at 2000 mg L^{-1} in ultrapure water: 302 ± 31.37 nm and − 14.13 mV	MTI Corporation	*D. magna*	ISO 6341	Concentrations tested: 3.6-2000 mg L^{-1}<br><br>ISO medium	Immobilization	48-h EC_{50} = 22 ± 0.7 mg CuO L^{-1}	[6]
CuO coated with Polyaniline	50-100 nm supplied by transmission electron microscopy (TEM)	Synthesized	*D. magna*	ISO 6341	Concentrations tested: 0.12-125 mg L^{-1}<br><br>ISO medium	Immobilization	48-h EC_{50} = 0.48 mg CuO L^{-1}	[7]
CuO	400 nm supplied by TEM	Synthesized	*Landoltia punctata*	OECD 221; ISO 20079	Concentrations tested: 0.1-10 g L^{-1}<br><br>Freshwater inorganic medium by OECD<br><br>DF: 10	Dry weight	7-days LOEC = 10 g CuO L^{-1}	[8]
CuO	30 nm primary size	Alfa Aesar	*R. subcapitata*	OECD 201	Maximum concentration tested: 6.4 mg Cu L^{-1}<br>Concentration expressed in terms of metal and not metal oxide content<br><br>Algal growth medium OECD	Growth rate	72-h EC50 = 0.71(0.5-1.9) mg Cu L^{-1}	[9]

Table S2. Physicochemical characteristics and toxic effects of different nano-ZnO on freshwater species. The primary particle size was supported by the manufacturer. NR: data not reported.

NM	Physicochemical Characterization	Origin	Freshwater species	Standard Test Protocol	Range concentrations tested, testing medium and dilution factor (DF)	Parameters	Endpoints - ECx (95% confidence intervals when available), NOEC and LOEC	References
ZnO	25 nm primary size; Hydrodynamic size, PI and ZP, respectively, measured at 20 mg L^{-1} in MBL medium: 178.61 ± 116.61 nm, 0.65 and − 27.5 ± 1.12 mV.	PlasmaChem	*R. subcapitata*	OECD 201	Concentrations tested: 1.3-5 mg L^{-1}<br><br>Woods Hole MBL medium<br><br>DF: 1.4	Growth rate	72-h EC_{50} = 4.86 (4.81-4.91) mg ZnO L^{-}1; LOEC: 3.6 mg L^{-1}	Present work
ZnO	25 nm primary size; Hydrodynamic size, PI and ZP, respectively, measured at 20 mg L^{-1} in ASTM medium: 281.80 ± 214.85 nm, 0.76 and − 2.25 ± 0.16 mV	PlasmChem	*D. magna*	OECD 202	Concentrations tested: 0.28-1.9 mg L^{-1}<br><br>ASTM medium<br><br>DF: 1.60	Immobilization	48-h LC_{50} = 1.33 mg ZnO L^{-1}	Present work
ZnO	25 nm primary size; Hydrodynamic size, PI and ZP, respectively, measured at 20 mg L^{-1} in Steinberg medium: >	PlasmChem	*L. minor*	OECD 221	Concentrations tested: 1.9-20 mg L^{-1}<br><br>Steinberg medium<br><br>DF: 1.60	Growth rate (Frond number and dry weight)	7-days LOEC = 1.9 mg ZnO L^{-1} (frond number and dry weight)	Present work

	1000 nm, 1.0 and − 10.20± 1.22 mV							
ZnO	10-15 nm primary size; Hydrodynamic size, PI and ZP, respectively, measured at 10 mg L^{-1} in AFW medium after 48h: 3855 ± 1807 nm, 0.86 ± 0.25 and − 4.10 ± 0.14 mV	Nanogate	*D. magna*	OECD 202	Concentrations tested: NR<br><br>OECD artificial freshwater (AFW)	Immobilization	48-h EC_{50} = 1.9 (1.7-2.2) mg Zn L^{-1}	[1]
ZnO	10-15 nm primary size; Hydrodynamic size, PI and ZP, respectively, measured at 10 mg L^{-1} in Lake Raku waters after 48h: 1135 ± 55 nm, 0.56 ± 0.11 and − 15.00 ± 0.25 mV	Nanogate	*D. magna*	OECD 202	Concentrations tested: NR<br><br>Lake Raku waters	Immobilization	48-h EC_{50} = 0.5 (0.4-0.5) mg Zn L^{-1}	[1]
ZnO	10-15 nm primary size; Hydrodynamic size, PI and ZP, respectively, measured at 10 mg L^{-1} in Lake Ülemiste waters after 48h: 445 ± 17 nm, 0.37 ± 0.029 and – 16.0 ± 0.21 mV	Nanogate	*D. magna*	OECD 202	Concentrations tested: NR<br><br>Lake Ülemiste waters	Immobilization	48-h EC_{50} = 0.7 (0.5-0.9) mg Zn L^{-1}	[1]

ZnO	10-15 nm primary size; Hydrodynamic size, PI and ZP, respectively, measured at 10 mg L^{-1} in MHW medium after 6 days: 3385 ± 310 nm, 0.30 ± 0.13 and – 5.0 ± 0.3 mV	Nanogate	*H. incongruens*	OSTRAC ODTOX KIT F (based on ISO 14371)	Concentrations tested: NR<br><br>USEPA artificial freshwater with algae addition (MHW)	Mortality	6-days LC_{50} = 0.3 (0.3-0.5) mg Zn L^{-1}	[1]
ZnO	10-15 nm primary size; Hydrodynamic size, PI and ZP, respectively, measured at 10 mg L^{-1} in Lake Raku waters after 6 days: 1052 ± 119 nm, 0.46 ± 0.036 and – 15.00 ± 0.49 mV	Nanogate	*H. incongruens*	OSTRAC ODTOX KIT F (based on ISO 14371)	Concentrations tested: NR<br><br>Lake Raku waters	Mortality	6-days LC_{50} = 0.5 (0.3-0.6) mg Zn L^{-1}	[1]
ZnO	10-15 nm primary size; Hydrodynamic size, PI and ZP, respectively, measured at 10 mg L^{-1} in Lake Ülemiste waters after 6 days: 445 ± 17 nm, 0.37 ± 0.029 and – 16.00 ± 0.21 mV	Nanogate	*H. incongruens*	OSTRAC ODTOX KIT F (based on ISO 14371)	Concentrations tested: NR<br><br>Lake Raku Ülemiste	Mortality	6-days LC_{50} = 0.6 (0.5-0.7) mg Zn L^{-1}	[1]
ZnO	< 100 nm primary size	Commercial (Sigma-Aldrich)	*D. pulex*	USEPA-821-R-02-012	Concentrations tested: 0, 0.15, 0.3, 0.6, 1.2, 2.4, 4.8, and 9.6 mg L^{-1}	Immobilization	48-h EC_{50} = 0.19 (0.16-0.23) mg Zn L^{-1}	[10]

					Reconstituted moderately hard water (RMHW)			
ZnO functionalized with 3-[2-(2-aminoethylamino)ethylamino]propyl-trimethoxysilane (AF)	ZnO: Size of 32 nm (supplied by TEM). Hydrodynamic size and ZP, respectively, measured at 500 mg L^{-1} in M4 culture medium: 849.83 ± 36.88 nm and 16.62 ± 0.66 mV; ZnO-AF: Size of 26 nm (supplied by TEM). Hydrodynamic size and ZP, respectively, measured at 500 mg L^{-1}in M4 culture medium: 1827.31 ± 250.97 nm and 6.24 ± 0.22 mV	Synthesized	*D. magna*	ISO 6341	Concentrations tested: 1.3-5.2 mg L^{-1} M4 culture medium	Immobilization	ZnO-48-h EC_{50} = 2.67 ± 0.19 mg ZnO L^{-1} ZnO-AF-48-h EC_{50} = 3.12 ± 0.40 mg ZnO L^{-1}	[11]
ZnOfunctionalized with 3-[2-(2-aminoethylamino)ethylamino]propyl-	ZnO: Size of 32 nm (supplied by TEM). Hydrodynamic size and ZP, respectively, measured at 500 mg L^{-1} in M4 culture medium:	Synthesized	*D. magna*	ISO 10706; OECD 211	Concentrations tested: 0.29-0.98 mg L^{-1} M4 culture medium	Mortality and Reproduction	ZnO: LOEC for reproduction = 0.98 mg ZnO L^{-1}; ZnO-AF: LOEC for survival= 0.98 mg ZnO L^{-1}; LOEC for reproduction = 0.65 mg ZnO L^{-1}	[11]

trimethoxysilane (AF)	849.83 ± 36.88 nm and 16.62 ± 0.66 mV; ZnO-AF: Size of 26 nm (supplied by TEM). Hydrodynamic size and ZP, respectively, measured at 500 mg L^{-1} in M4 culture medium: 1827.31 ± 250.97 nm and 6.24 ± 0.22 mV							
ZnO	< 100 nm primary size	Sigma-Aldrich	*Scenedesmus rubescens*	OECD 201	Concentrations tested: 0.81-810 mg L^{-1}<br><br>1/3 N BG-11 medium<br><br>Bolds Basal medium<br><br>DF: 10	Growth rate	28-days IC_{50} = 8.93 mg ZnO L^{-1} (in 1/3 N BG-11 medium)<br><br>28-day IC_{50} > 810 mg ZnO L^{-1} (in Bolds Basal medium)	[12]
ZnO	20-30 nm primary size; Hydrodynamic size measured at 100 mg L^{-1} in MWH culture medium after 48h: > 5000 nm	Alfa Aesar	*D. magna*	OECD 202	Maximum concentration tested: 10 mg L^{-1}<br><br>MHW medium	Immobilization	48-h EC_{50} = 1.4 ± 0.3 mg ZnO L^{-1}	[4]
ZnO	< 50 nm primary size; Hydrodynamic size and ZP, respectively, measured at 0.1 mg L^{-1}	Sigma-Aldrich	*D. magna*	OECD 202	Concentrations tested: 0, 0.10, 0.33, 1.00, 3.30 and 10.00 mg L^{-1}<br><br>Mineral water	Immobilization	48-h EC_{50} = 1.9 (1.5-2.4) mg ZnO L^{-1}	[13]

	in mineral water after 1h: high particle agglomeration and −10.50 ± 0.32 mV						48-h EC_{50} = 1.5 (1.2-1.9) mg Zn L^{-1}	
ZnO	< 100 nm primary size; Hydrodynamic size and ZP, respectively, measured at 0.1 mg L^{-1} in mineral water after 1h: high particle agglomeration and −9.81 ± 0.49 mV	Sigma-Aldrich	*D. magna*	OECD 202	Concentrations tested: 0, 0.10, 0.33, 1.00, 3.30 and 10.00 mg L^{-1}<br><br>Mineral water	Immobilization	48-h EC_{50} = 3.1 (2.4-4.0) mg ZnO L^{-1}<br><br>48-h EC_{50} = 2.5 (1.9-3.2) mg Zn L^{-1}	[13]
ZnO	20 nm primary size	Nanjing High Technology NANO Co., LTD	*D. magna*	OECD 202	Concentrations tested: 0, 0.01, 0.05, 0.10, 0.50, 1.00 and 5.00 mg L^{-1}<br><br>Reconstituted water (OECD 202)	Immobilization	48-h LC_{50} = 0.622 (0.411-0.805) mg ZnO L^{-1}	[14]
ZnO	70 nm primary size	Sigma-Aldrich	*D. magna*	Daphtoxkit F™	Maximum concentration tested: 10 mg Zn L^{-1}	Mortality	48-h LC_{50} = 3.2 ± 1.3 mg Zn L^{-1}	[15]
ZnO	70 nm primary size	Sigma-Aldrich	*Thamnocephalus platyurus*	ThamnotoxkitF™	Maximum concentration tested: 1.0 mg Zn L^{-1}	Mortality	24-h LC_{50} = 0.18 ± 0.03 mg Zn L^{-1}	[15]

Table S3. Physicochemical characteristics and toxic effects of different nano-TiO_2 on freshwater species. The primary particle size was supported by the manufacturer.

NM	Physicochemical Characterization	Origin	Freshwater species	Standard Test Protocol	Range concentrations tested, testing medium and dilution factor (DF)	Parameters	Endpoints - ECx (95% confidence intervals when available), NOEC and LOEC	References
Hydrophilic TiO_2 powder stabilized by HNO_3	Anatase, 4-8 nm primary size; Hydrodynamic size, PI and ZP, respectively, measured at 20 mg L^{-1} in MBL medium: 770.54 ± 174.84 nm, 0.92 and – 21.6 ± 0.80 mV	PlasmaChem	*R. subcapitata*	OECD 201	Concentrations tested: 1.9-20 mg L^{-1}<br>Woods Hole MBL medium<br>DF: 1.6	Growth rate	72-h LOEC = 20 mg TiO_2 L^{-1}	Present work
Hydrophilic TiO_2 powder stabilized by HNO_3	Anatase, 4-8 nm primary size; Hydrodynamic size and ZP, respectively, measured at 20 mg L^{-1} in ASTM medium: high particle agglomeration and – 2.25 ± 0.16 mV	PlasmaChem	*D. magna*	OECD 202	Concentrations tested: 1.9-20 mg L^{-1}<br>ASTM medium<br>DF: 1.6	Immobilization	48-h LC_{50} > 20 mg TiO_2 L^{-1}	Present work
Hydrophilic TiO_2 powder stabilized by HNO_3	Anatase, 4-8 nm primary size; Hydrodynamic size, PI and ZP, respectively, measured at 20 mg L^{-1} in Steinberg medium: 899.71 ± 75.90 nm, 0.92 and -4.33 ± 0.72	PlasmaChem	*L. minor*	OECD 221	Concentrations tested: 1.9-20 mg L^{-1}<br>Steinberg medium<br>DF: 1.6	Growth rate	7-days LOEC = 12.5 mg TiO_2 L^{-1}	Present work

TiO_2	Anatase/rutile, 21 nm primary size; Hydrodynamic size and PI, respectively measured at 2.00 g $nTiO_2$ L^{-1}: 59.60 ± 3.78 nm and 0.14	Aeroxide P25, Evonik	*D. magna*	OECD 202	Concentrations tested: 0.10, 0.20, 1.0, 2.0. 4.0, 8.0, 16.0, 32.0 and 64 mg L^{-1}<br><br>ASTM medium	Immobilization	96-h EC_{50} = 28.83 (13.30–44.37) mg TiO_2 L^{-1}	[16]
TiO_2	82% anatase/18% rutile, 21 nm primary size; Hydrodynamic size, PI and ZP, respectively, measured at 1600 mg L^{-1} in reconstituted water: 1079 nm, 0.425-0492 and −15.77 ± 0.25 mV	Sigma-Aldrich	*C. silvestrii*	ABNT NBR 12713<br><br>OECD 202	Concentrations tested: 6.25 -1600 mg L^{-1}<br><br>Reconstituted water<br><br>DF: 2	Immobilization	48-h EC_{50} = 48.70 to 96.59 mg TiO_2 L^{-1}	[17]
TiO_2	Anatase, 20 nm primary size; Hydrodynamic diameter and ZP, respectively, measured at 100 mg L^{-1} in TAP medium: 367.23 ± 16.15 nm and -18.11 ± 0.23 mV	Shanghai Chaowei nanotechnology Co.	*C. reinhardtii*	OECD 201	Concentrations tested: 50, 100, 200, 300 and 400 mg L^{-1}<br><br>TAP medium	Growth rate	96-h EC_{50} = 359.822 mg TiO_2 L^{-1}	[18]
TiO_2	Anatase, ~5 nm primary size; Hydrodynamic size and ZP, respectively, measured at 100 mg L^{-1}	unknown	*D. magna*	ISO 6341:2014	Concentrations tested: 0, 1.0, 10.0 and 100 mg L^{-1}<br><br>ISO dilution water	Immobilization	LOEC = 10 mg TiO_2 L^{-1}	[19]

	in ISO dilution water: 375 nm and -17.0 ± 2.4 mV							
TiO_2	Anatase, < 25 nm primary size; Hydrodynamic size measured at 75 µM in sterile lake water: 646.53 ± 28.64 nm	Sigma-Aldrich	*Scenedesmus obliquus*	OECD 201	25-800 µM<br><br>Sterile lake water<br><br>DF: 2	Growth rate	72-h EC_{50} = 136.88 ± 2.30 µM	[20]
TiO_2	79% anatase/21% rutile, <100 nm primary size; Size of 38±12 nm (supplied by TEM); Hydrodynamic size PI and ZP respectively, measured at 1 mg L^{-1} in ultrapure water: 176.9 ± 62 nm, 0.08 and -27.1±3.9 mV; Hydrodynamic size, PI and ZP, respectively, measured at 1 mg L^{-1} in artificial freshwater: 591.3±75.2 nm, 0.594 and -16.9±4.1 mV.	Sigma-Aldrich	*C. reinhardtii*	OECD 201	Concentrations tested: 0.1, 1.0, 10.0, 100.0, 200.0 and 400 mg L^{-1} under continuous white light;<br><br>Concentrations tested: 0.1, 1.0, 2.0, 5.0 and 10 mg L^{-1} under the visible light plus UV-A treatment<br><br>Artificial freshwater	Growth rate	UV-A + Visible light<br><br>72-h EC_{50} = 2.30 ± 1.76 mg TiO_2 L^{-1}<br><br>Visible light only<br><br>72-h EC_{50} = 551.7 ± 163.79 mg TiO_2 L^{-1}	[21]
TiO_2	Anatase, < 25 nm primary size;	Sigma-Aldrich	*Ceriodaphnia dubia*	OECD 202	Concentrations tested: 2 - 128 mg L^{-1}<br><br>Sterile lake water	Immobilization	Under visible irradiation	[22]

	Size of 9.5 ± 1 nm (supplied by TEM); Hydrodynamic size and PI, respectively, measured in deionized water: 307.07 ± 37.9 nm and 0.14				DF: 2		48-h LC_{50} = 37.04±1.93 mg TiO_2 L^{-1}<br><br>Under UV-A irradiation<br><br>48-h LC_{50} = 22.56±0.54 mg TiO_2 L^{-1}	
TiO_2	Rutile, < 100 nm primary size; Size of 26 ± 3 nm in length and 4 ± 0.5 nm in breadth (rod-shaped) (supplied by TEM); Hydrodynamic size and PI, respectively, measured in deionized water: 218.92 ± 1.92 nm and 0.25	Sigma-Aldrich	*Ceriodaphnia dubia*	OECD 202	Concentrations tested: 2 - 128 mg L^{-1}<br><br>Sterile lake water<br><br>DF: 2	Immobilization	Under visible irradiation<br><br>48-h LC_{50} = 48 ± 3.64 mg TiO_2 L^{-1}<br><br>Under UV-A irradiation<br><br>48-h LC_{50} = 23.76 ± 0.57 mg TiO_2 L^{-1}	[22]
TiO_2	Anatase/rutile, 21 nm primary size; Hydrodynamic size at 1 mg L^{-1} after 72 h in lake freshwater in dark conditions: 395.4 ± 4.13 nm; in visible light: 397.3	Aeroxide P25, Sigma-Aldrich	*Chlorella sp*	OECD 201	Concentrations tested: 0.25 - 64 mg L^{-1}<br><br>Sterilized freshwater medium<br><br>DF: 2	Growth rate	Dark<br>*72-h EC_{50} = 5.95 ± 0.18 mg* TiO_2 *L^{-1}*<br><br>Visible light<br>*72-h EC_{50} = 2.16 ± 0.06 mg* TiO_2 *L^{-1}*	[23]

	± 6.78 nm and in UV-A: 395.6 ± 8.5 nm						UV-A<br>*72-h EC_{50}* = 1.565 ± 0.04 mg TiO_2 L^{-1}	
TiO_2	Anatase/rutile, 21 nm primary size; Hydrodynamic size at 1 mg L^{-1} after 72 h in lake freshwater in dark conditions: 395.4 ± 4.13 nm; in visible light: 397.3 ± 6.78 nm and in UV-A: 395.6 ± 8.5 nm	Aeroxide P25, Sigma-Aldrich	*Scenedesmus sp*	OECD 201	Concentrations tested: 0.25 - 64 mg L^{-1}<br><br>Sterilized freshwater medium<br><br>DF: 2	Growth rate	Dark<br>*72-h EC_{50} = 7.632 ± 0.13 mg* TiO_2 *L^{-1}*<br><br>Visible light<br>*72-h EC_{50} = 4.139 ± 0.11 mg* TiO_2 *L^{-1}*<br><br>UV-A<br>*72-h EC_{50} = 2.752 ± 0.08 mg* TiO_2 *L^{-1}*	[23]
TiO_2	HOMBIKAT UV100 (NM-101)<br>Anatase, < 10 nm primary size;<br>Hydrodynamic size, PI and ZP, respectively, measured at 50 mg L^{-1} in OECD algae growth medium: 763 ± 15 nm, 0.27 and -18 ± 0.7 mV	Provided by European Commission Joint Research Centre within the OECD sponsorship program	*R. subcapitata*	OECD 201	Concentrations tested: 2.1 - 50 mg L^{-1}<br><br>OECD algal growth medium	Growth rate	24-well microplates<br>72-h EC_{50} = 8.5 (5-16) mg TiO_2 L^{-1}<br>Cylindrical vials<br><br>72-h EC_{50} = 2.7 (1-5) mg TiO_2 L^{-1}<br>Erlenmeyer flasks<br>72-h EC_{50} > 50 mg L^{-1}	[24]

	UV-Titan m212 (NM-104)<br>Rutile, 20 nm, coating Al_2O_3 (supported by the manufacturer); Z-Average, PDI and ZP in OECD algae growth medium (50 mg L^{-1} TiO_2), 1224 ± 126 nm, 0.25 and 12 ± 0.6 mV	Provided by European Commission Joint Research Centre within the OECD sponsorship program	*R. subcapitata*	OECD 201	Concentrations tested: 2.1 - 50 mg L^{-1}<br>OECD algal growth medium	Growth rate	UV-Titan m212<br>24-well microplates<br>72-h EC_{50} > 50 mg TiO_2 L^{-1}<br>Cylindrical vials<br>72-h EC_{50} = 39 (32-47) mg TiO_2 L^{-1}<br>Erlenmeyer flasks<br>72-h EC_{50} > 50 mg L^{-1}	[24]
TiO_2	Anatase, <25 nm primary size;<br>Hydrodynamic size, PI and ZP, respectively, measured at 20 mg L^{-1} in MBL medium: 1225 ± 26 nm, 0.69 and -22 ± 4 mV	Sigma-Aldrich	*R. subcapitata*	OECD 201	Concentrations tested: 8.2 – 20.0 mg L^{-1}<br>Woods Hole MBL<br>DF: 1.25	Growth rate	72-h EC_{50} > 20 mg TiO_2 L^{-1}	[25]
TiO_2	Anatase, <25 nm primary size;<br>Hydrodynamic size, PI and ZP, respectively, measured at 20 mg L^{-1} in Steinberg medium: 1605 ± 163 nm, 0.94 and -14.9 ± 3.7 mV	Sigma-Aldrich	*L. minor*	OECD 221	Concentrations tested: 8.2 – 20.0 mg L^{-1}<br>Steinberg medium<br>DF: 1.25	Growth rate	7-days EC_{50} > 20 mg TiO_2 L^{-1}	[25]

TiO_2	Anatase, <25 nm primary size;<br>Hydrodynamic size, PI and ZP, respectively, measured at 20 mg L^{-1} in ASTM medium: 2271 ± 230 nm, 0.81 and -19.3 ± 4 mV	Sigma-Aldrich	*D. magna*	OECD 202<br>OECD 211	Concentrations tested: 8.2 – 20.0 mg L^{-1}<br><br>ASTM medium<br><br>DF: 1.25	Immobilization<br><br>Reproduction	Immobilization *D. magna*<br>48-h EC_{50} > 20 mg TiO_2 L^{-1}<br><br>Reproduction *D. magna*<br>LOEC (21-days) = 16 mg TiO_2 L^{-1}	[25]
TiO_2	Anatase/rutile, 21 nm primary size;<br>Size of 34.10 ± 2.70 nm (supplied by TEM);<br>Hydrodynamic size and ZP, respectively, measured in deionized water: 190.5 ± 3.4 nm and -13.9 mV	Aeroxide P25, Sigma-Aldrich	*Lymnea luteola*	OECD 203	Concentrations tested: 5, 15, 30, 60, 120 and 200 mg L^{-1}<br><br>Test water (no more specifications)	Mortality	96-h LC_{50} = 112 mg TiO_2 L^{-1}	[26]
TiO_2	''TA''- anatase, < 25 nm primary size;<br>Hydrodynamic size, PI and ZP, respectively, measured at 100 mg L^{-1} in culture medium after 24 h: 578.2 ± 52.9 nm, 0.4, -21.5 ± 0.7 mV	("TA" from Sigma-Aldrich	*D. similis*	USEPA-EPA-540/9-85-009	Concentrations tested:<br>Under visible light<br>100 and 1000 mg L^{-1} of both nano-TiO_2 formulations.<br><br>Under UV light 6.25 - 100 mg L^{-1} of TM, and 62.5 -1000 mg L^{-1} of TA.<br><br>*D. similis* culture medium (no more specifications)	Immobilization	"TA"<br>Visible light: 48-h EC_{50} > 1000 mg TiO_2 L^{-1}<br>UV light: 48-h EC_{50} = 750.55 (599.56–1008.92) mg TiO_2 L^{-1}<br><br>"TM"<br>Visible light: 48-h EC_{50} > 1000 mg TiO_2 L^{-1}	[27]

	‘‘TM”- 20 % rutile/80 % anatase, 25 nm (primary size supported by the manufacturer); Z-average, PDI and ZP in culture medium (100 mg L^{-1} TiO_2 after 24 h), 1373.3 ± 360.2 nm, 0.9, -13.3 ± 2.0 mV.	“TM” Aeroxide P25 from Evonik					UV light: 48-h EC_{50} = 60.16 (48.30–77.94) mg TiO_2 L^{-1}	
TiO_2	Anatase, < 25 nm primary size	Sigma-Aldrich	*C. dubia*	OECD 202	1.0 - 64 mg L^{-1}<br><br>DF: 2	Immobilization	Under visible light<br>48-h EC_{50} = 8.26 mg TiO_2 L^{-1}<br><br>Dark<br>48-h EC_{50} = 27.45 mg TiO_2 L^{-1}	[28]
TiO_2	Anatase/rutile; Hydrodynamic size measured at 20 mg L^{-1} in ISO test water: 968 nm; Hydrodynamic size measured at 20 mg L^{-1} in river water: 605 nm; ZP ranged between -10.3 and -14.9 mV	Degussa P25, Evonik	*D. magna*	OECD 202	Under dark<br>10 - 105 mg L^{-1}<br><br>UV-A light<br>1.0 – 10.5 mg L^{-1}<br><br>ISO test water<br><br>River water<br><br>DF: 1.8	Immobilization	ISO test water<br>Dark 48-h EC_{50} = 29.7 (16.83–53) mg TiO_2 L^{-1}<br>UV-A 48-h EC_{50} = 1.2 (0.73–2.0) mg TiO_2 L^{-1}<br><br>River water<br>Dark 48-h EC_{50} = 33.6 (22.83–44.4) mg TiO_2 L^{-1}<br>UV-A 48-h EC_{50} = 3.4 (1.63–7.4) mg TiO_2 L^{-1}	[29]

TiO_2	Anatase, < 25 nm primary size; Size of 20 nm (supplied by TEM); Hydrodynamic size in algal medium 198.1 ± 1.6 nm	Sigma-Aldrich	*Scenedesmus sp.*	OECD 201	3.0 - 192 mg L^{-1} Bold Basal medium DF: 2	Growth rate	72-h EC_{50} = 21.2 mg TiO_2 L^{-1}	[30]
TiO_2	Anatase, < 25 nm primary size; Size of 20 nm (supplied by TEM); Hydrodynamic size in algal medium 198.1 ± 1.6 nm	Sigma-Aldrich	*Chlorella sp.*	OECD 201	3.0 - 192 mg L^{-1} Bold Basal medium DF: 2	Growth rate	72-h EC_{50} = 16.12 mg TiO_2 L^{-1}	[30]
TiO_2	Anatase, 6 nm primary size; Hydrodynamic size and PI, respectively, measured of the stock suspension: 100 nm and 0.15	Crenox GmbH	*D. magna*	OECD 202	0.5 – 8.0 mg L^{-1} ISO test medium DF: 2	Immobilization	72-h EC_{50} = 3.8 mg TiO_2 L^{-1} 96-h EC_{50} = 0.73 mg TiO_2 L^{-1}	[31]

Table S4. Physicochemical characteristics and toxic effects of different nano-SiO_2 on freshwater species. The primary particle size was supported by the manufacturer.

NM	Physicochemical Characterization	Origin	Freshwater species	Standard Test Protocol	Range concentrations tested, testing medium and dilution factor (DF)	Parameters	Endpoints - ECx (95% confidence intervals when available), NOEC and LOEC	References
Hydrophilic SiO_2	7-14 nm (primary size; Hydrodynamic size, PI and ZP, respectively, measured at 20 mg L^{-1} in MBL medium: 209.20 ± 179.96 nm, 0.86 and -27.3 ± 3.81 mV	PlasmaChem	*R. subcapitata*	OECD 201	Concentrations tested:1.9-20 mg L^{-1}<br>Woods Hole MBL medium<br>DF: 1.6	Growth rate	72-h LOEC = 7.8 mg SiO_2 L^{-1}	Present work
Hydrophilic SiO_2	7-14 nm (primary size; Hydrodynamic size, PI and ZP, respectively, measured at 20 mg L^{-1} in ASTM medium: 235.18 ± 104.19, 0.44, − 15.3 ± 1.08 mV;	PlasmaChem	*D. magna*	OECD 202	Concentrations tested: 1.9-20 mg L^{-1}<br>ASTM medium<br>DF: 1.6	Immobilization	48-h LC_{50} > 20 mg SiO_2 L^{-1}	Present work
Hydrophilic SiO_2	7-14 nm (primary size; Hydrodynamic size, PI and ZP, respectively, measured at 20 mg L^{-1} in Steinberg medium: 195.49 ± 85.65 nm, 0.43 and -8.39 ± 1.80 mV	PlasmaChem	*L. minor*	OECD 221	Concentrations tested:1.9-20 mg L^{-1}<br>Steinberg<br>DF: 1.6	Growth rate	7-days EC_{50} > 20 mg SiO_2 L^{-1}	Present work

SiO_2 aqueous suspension 30%	10 nm primary size; Hydrodynamic size, PI and ZP, respectively, measured at 20 mg L^{-1} in MBL medium: 11.77 ± 2.63 nm, 0.22 and − 25.7 ± 2.0 mV;	PlasmaChem	*R. subcapitata*	OECD 201	Concentrations tested:1.9-20 mg L^{-1}<br>MBL<br>DF: 1.6	Growth rate	72-h LOEC = 1.9 mg SiO_2 L^{-1}	Present work
SiO_2 aqueous suspension 30%	Hydrodynamic size, PI and ZP, respectively, measured at 20 mg L^{-1} in ASTM medium: 12.51 ± 1.65, 0.13, − 13.9 ± 1.39 mV;	PlasmaChem	*D. magna*	OECD 202	Concentrations tested:1.9-20 mg L^{-1}<br>ASTM<br>DF: 1.6	Immobilization	48-h LC_{50} > 20 mg SiO_2 L^{-1}	Present work
SiO_2 aqueous suspension 30%	Hydrodynamic size, PI and ZP, respectively, measured at 20 mg L^{-1} in Steinberg medium: 50.01 ± 26.03 nm, 0.52 and -9.82 ±0.67 mV	PlasmaChem	*L. minor*	OECD 221	Concentrations tested:1.9-20 mg L^{-1}<br>Steinberg<br>DF: 1.6	Growth rate	7-days EC_{50} > 20 mg SiO_2 L^{-1}	Present work
SiO_2	10-20 nm primary size; Hydrodynamic size and ZP, respectively, measured at 400 mg L^{-1} in TAP medium: 570.27 ± 27.83 nm and -0.55 ± 0.021 mV	Sigma-Aldrich	*C. reinhardtii*	OECD 201	Concentrations tested: 100, 200, 400, 600 and 800 mg L^{-1}<br>TAP medium	Growth rate	96-h EC_{50} = 1180.725 mg SiO_2 L^{-1}	[18]

SiO_2	Colloidal silica LUDOX LS which corresponds to 363 g SiO_2 L^{-1}; Hydrodynamic size and PI, respectively, measured in OECD medium: 12.5 ± 0.2 nm and 0.26 ± 0.02	Sigma-Aldrich	*R. subcapitata*	OECD 201	Concentrations tested: 2.2 - 460 mg L^{-1}<br><br>OECD medium	Growth rate	LOEC = 10.0 mg SiO_2 L^{-1}<br><br>72-h EC20 = 20.0 ± 5.0 mg L^{-1}	[32]
SiO_2	Colloidal silica LUDOX TM40 which corresponds to 521 g SiO_2 L^{-1}; Hydrodynamic size and PI, respectively, measured in OECD medium: 27.0 ± 0.5 nm and 0.17 ± 0.02	Sigma-Aldrich	*R. subcapitata*	OECD 201	Concentrations tested: 2.2 - 460 mg L^{-1}<br><br>OECD medium	Growth rate	LOEC = 10.0 mg SiO_2 L^{-1}<br><br>72-h EC20 = 28.8 ± 3.2 mg SiO_2 L^{-1}	[32]

References

1. Muna, M.; Blinova, I.; Kahru, A.; Vrček, I.V.; Pem, B.; Orupõld. K.; Heinlaan, M. Combined effects of test media and dietary algae on the toxicity of CuO and ZnO nanoparticles to freshwater microcrustaceans *Daphnia magna* and *Heterocypris incongruens*: Food for thought. *Nanomaterials* **2019**, *9*, 23.
2. Mansano, A.S.; Souza, J.P.; Cancino-Bernardi, J.; Venturini, F.P.; Marangoni, V.S.; Zucolotto, V. Toxicity of copper oxide nanoparticles to Neotropical species *Ceriodaphnia silvestrii* and *Hyphessobrycon eques*. *Environ. Pollut*. **2018**, *243*, 723–733.
3. Dolenc, K.J. Effects of exposure to nano and bulk sized TiO_2 and CuO in *Lemna minor*. *Plant. Physiol. Biochem*. **2017**, *119*, 43–49.
4. Kim, S.; Samanta, P.; Yoo, J.; Kim, W.-K.; Jung, J. Time-dependent toxicity responses in *Daphnia magna* exposed to CuO and ZnO nanoparticles. *Bull. Environ. Contam. Toxicol.* **2017**, *98*, 502–507.
5. Adam, N.; Vakurov, A.; Knapen, D.; Blust, R. The chronic toxicity of CuO nanoparticles and copper salt to *Daphnia magna*. *J. Hazard. Mater.* **2015**, *283*, 416–422.
6. Rossetto, A.L.d.O.F.; Melegari, S.P.; Ouriques, L.C.; Matias, W.G. Comparative evaluation of acute and chronic toxicities of CuO nanoparticles and bulk using *Daphnia magna* and *Vibrio fischeri*. *Sci. Total Environ*. **2014**, *490*, 807–814.
7. Rossetto, A.L.d.O.F.; Vicentini, D.S.; Costa, C.H.; Melegari, S.P.; Matias, W.G. Synthesis, characterization and toxicological evaluation of a core–shell copper oxide/polyaniline nanocomposite. *Chemosphere* **2014**, *108*, 107–114.
8. Lalau, C.M.; Mohedano, R.d.A.; Schmidt, É.C.; Bouzon, Z.L.; Ouriques, L.C.; dos Santos, R.W.; da Costa, C.H.; Vicentini, D.S.; Matias, W.G. Toxicological effects of copper oxide nanoparticles on the growth rate, photosynthetic pigment content, and cell morphology of the duckweed *Landoltia punctata*. *Protoplasma* **2015**, *252*, 221–229.
9. Aruoja, V.; Dubourguier, H.-C.; Kasemets, K.; Kahru, A. Toxicity of nanoparticles of CuO, ZnO and TiO_2 to microalgae *Pseudokirchneriella subcapitata*. *Sci. Total Environ.* **2009**, *407*, 1461–1468.
10. Lin, L.; Xu, M.; Mu, H.; Wang, W.; Sun, J.; He, J.; Qiu, J-W.; Luan, T. Quantitative proteomic analysis to understand the mechanisms of zinc oxide nanoparticle toxicity to *Daphnia pulex* (Crustacea: Daphniidae): Comparing with bulk zinc oxide and zinc salt. *Environ. Sci. Technol*. **2019**, *53*, 5436–5444.
11. Melegari, S.P.; Fuzinatto, C.F.; Gonçalves, R.A.; Oscar, B.V.; Vicentini, D.S.; Matias, W.G. Can the surface modification and/or morphology affect the ecotoxicity of zinc oxide nanomaterials? *Chemosphere* **2019**, *224*, 237–246.
12. Aravantinou, A.F.; Andreou, F.; Manariotis, I.D. Long-term toxicity of ZnO nanoparticles to *Scenedesmus rubescens* cultivated in different media. *Sci. Rep*. **2017**, *7*, 13454.
13. Santo, N.; Fascio, U.; Torres, F.; Guazzoni, N.; Tremolada, P.; Bettinetti, R.; Mantecca, P.; Bacchetta, R. Toxic effects and ultrastructural damages to *Daphnia magna* of two differently sized ZnO nanoparticles: Does size matter? *Water Res*. **2014**, *53*, 339–350.
14. Zhu, X.; Zhu, L.; Chen, Y.; Tian, S. Acute toxicities of six manufactured nanomaterial suspensions to *Daphnia magna*. *J. Nanoparticle Res*. **2009**, *11*, 67–75.
15. Heinlaan, M.; Ivask, A.; Blinova, I.; Dubourguier, H.-C.; Kahru, A. Toxicity of nanosized and bulk ZnO, CuO and TiO_2 to bacteria *Vibrio fischeri* and crustaceans *Daphnia magna* and *Thamnocephalus platyurus*. *Chemosphere* **2008**, *71*, 1308–1316.
16. Lüderwald, S.; Dackermann, V.; Seitz, F.; Adams, E.; Feckler, A.; Schilde, C.; Schulz, R.; Bundschuh, M. A blessing in disguise? Natural organic matter reduces the UV light-induced toxicity of nanoparticulate titanium dioxide. *Sci. Total Environ*. **2019**, *663*, 518–526.
17. de Lucca, G.M.; Freitas, E.C.; da Graça, G.M.M. Effects of TiO_2 nanoparticles on the neotropical cladoceran *Ceriodaphnia silvestrii* by waterborne and dietary routes. *Water Air Soil Pollut*. **2018**, *229*, 307.
18. Yu, Z.; Hao, R.; Zhang, L.; Zhu, Y. Effects of TiO_2, SiO_2, Ag and CdTe/CdS quantum dots nanoparticles on toxicity of cadmium towards *Chlamydomonas reinhardtii*. *Ecotoxicol. Environ. Saf.* **2018**, *156*, 75–86.
19. Novak, S.; Jemec Kokalj, A.; Hočevar, M.; Godec, M.; Drobne, D. The significance of nanomaterial post-exposure responses in *Daphnia magna* standard acute immobilisation assay: Example with testing TiO_2 nanoparticles. *Ecotoxicol. Environ. Saf.* **2018**, *152*, 61–66.
20. Iswarya, V.; Sharma, V.; Chandrasekaran, N.; Mukherjee, A. Impact of tetracycline on the toxic effects of titanium dioxide (TiO_2) nanoparticles towards the freshwater algal species, *Scenedesmus obliquus*. *Aquat. Toxicol*. **2017**, *193*, 168–177.

21. Sendra, M.; Moreno-Garrido, I.; Yeste, M.P.; Gatica, J.M.; Blasco, J. Toxicity of TiO_2, in nanoparticle or bulk form to freshwater and marine microalgae under visible light and UV-A radiation. *Environ. Pollut*. **2017**, *227*, 39–48.
22. Iswarya, V.; Bhuvaneshwari, M.; Chandrasekaran, N.; Mukherjee, A. Individual and binary toxicity of anatase and rutile nanoparticles towards *Ceriodaphnia dubia*. *Aquat. Toxicol.* **2016**, *178*, 209–221.
23. Roy, R.; Parashar, A.; Bhuvaneshwari, M.; Chandrasekaran, N.; Mukherjee, A. Differential effects of P25 TiO_2 nanoparticles on freshwater green microalgae: *Chlorella* and *Scenedesmus* species. *Aquat. Toxicol.* **2016**, *176*, 161–171.
24. Nicolas, M.; Séverine, L.M.; Anne, B.-N.; Pascal, P. Effect of two TiO_2 nanoparticles on the growth of unicellular green algae using the OECD 201 test guideline: Influence of the exposure system Manier. *Toxicol. Environ. Chem*. **2016**, *98*, 860–876.
25. Nogueira, V.; Lopes, I.; Rocha-Santos, T.A.P.; Rasteiro, M.G.; Abrantes, N.; Gonçalves, F.; Soares, A.M.V.M.; Duarte, A.C.; Pereira, R. Assessing the ecotoxicity of metal nano-oxides with potential for wastewater treatment. *Environ. Sci. Pollut. Res*. **2015**, *22*, 13212–1324.
26. Ali, D.; Ali, H.; Alarifi, S.; Kumar, S.; Serajuddin, M.; Mashih, A.P.; Ahmed, M.; Khan, M.; Adil, S.F.; Shaik, M.R.; Ansari, A. Impairment of DNA in a freshwater gastropod (*Lymnea luteola* L.) after exposure to titanium dioxide nanoparticles. *Arch. Environ. Contam. Toxicol*. **2015**, *68*, 543–552.
27. Clemente, Z.; Castro, V.L.; Jonsson, C.M.; Fraceto, L.F. Minimal levels of ultraviolet light enhance the toxicity of TiO_2 nanoparticles to two representative organisms of aquatic systems. *J. Nanoparticle Res*. **2014**, *16*, 2559.
28. Dalai, S.; Pakrashi, S.; Chandrasekaran, N.; Mukherjee, A. Acute Toxicity of TiO_2 nanoparticles to *Ceriodaphnia dubia* under visible light and dark conditions in a freshwater system. *PLoS ONE* **2013**, *8*, e62970.
29. Amiano, I.; Olabarrieta, J.; Vitorica, J.; Zorita, S. Acute toxicity of nanosized TiO_2 to *Daphnia magna* under UVA irradiation. *Environ. Toxicol. Chem.* **2012**, 31 (11), 2564–66.
30. Sadiq, I.M.; Dalai, S.; Chandrasekaran, N.; Mukherjee, A. Ecotoxicity study of titania (TiO_2) NPs on two microalgae species: *Scenedesmus* sp. and *Chlorella* sp. *Ecotoxicol. Environ. Saf.* **2011**; *74*, 1180–1187.
31. Dabrunz, A.; Duester, L.; Prasse, C.; Seitz, F.; Rosenfeldt, R.; Schilde, C.; Schaumann G.E.; Schulz, R. Biological surface coating and molting inhibition as mechanisms of TiO_2 nanoparticle toxicity in *Daphnia magna*. *PLoS ONE* **2011**, *6*, 1–7.
32. Van Hoecke, K.; De Schamphelaere, K.A.C.; Van der Meeren, P.; Lcucas, S.; Janssen, C.R. Ecotoxicity of silica nanoparticles to the green alga *pseudokirchneriella subcapitata*: Importance of surface area. *Environ. Toxicol. Chem*. **2009**, *27*, 1948–1957.

© 2020 by the authors. Licensee MDPI, Basel, Switzerland. This article is an open access article distributed under the terms and conditions of the Creative Commons Attribution (CC BY) license (http://creativecommons.org/licenses/by/4.0/).
